# Design and Synthesis of Multi-Functional Ligands through Hantzsch Reaction: Targeting Ca^2+^ Channels, Activating Nrf2 and Possessing Cathepsin S Inhibitory, and Antioxidant Properties

**DOI:** 10.3390/pharmaceutics16010121

**Published:** 2024-01-17

**Authors:** Irene Pachón-Angona, Paul J. Bernard, Alexey Simakov, Maciej Maj, Krzysztof Jozwiak, Anna Novotna, Carina Lemke, Michael Gütschow, Helene Martin, María-Jesús Oset-Gasque, José-Marco Contelles, Lhassane Ismaili

**Affiliations:** 1 Université de Franche-Comté, UMR INSERM 1322 LINC, F-25000 Besançon, France; pachon.angona.irene@gmail.com (I.P.-A.); paul.bernard@univ-fcomte.fr (P.J.B.); 2 Department of Biochemistry and Molecular Biology, Faculty of Pharmacy, Complutense University of Madrid, Plaza Ramón y Cajal s/n, Ciudad Universitaria, 28040 Madrid, Spain; mjoset@ucm.es; 3 Université de Franche-Comté, EFS, INSERM, UMR RIGHT, F-25000 Besançon, France; aleksei.simakov@univ-fcomte.fr (A.S.); helene.martin@univ-fcomte.fr (H.M.); 4 Department of Biopharmacy, Medical University of Lublin, ul. W. Chodzki 4a, 20-093 Lublin, Poland; maciej.maj@umlub.pl (M.M.); krzysztof.jozwiak@umlub.pl (K.J.); 5 Pharmaceutical Institut, An der Immenburg 4, D-53121 Bonn, Germany; novo.anna21@gmail.com (A.N.); wirtzcarina01@gmail.com (C.L.); guetschow@uni-bonn.de (M.G.); 6 Instituto Universitario de Investigación en Neuroquímica, Complutense University of Madrid, Ciudad Universitaria, 28040 Madrid, Spain; 7 Laboratory of Medicinal Chemistry (IQOG, CSIC) C/Juan de la Cierva 3, 28006 Madrid, Spain; 8 Center for Biomedical Network Research on Rare Diseases (CIBERER), CIBER, ISCIII, 28006 Madrid, Spain

**Keywords:** calcium channel inhibitors, proteases inhibitors, antioxidant response element, ORAC, Alzheimer’s disease

## Abstract

This work relates to the design and synthesis of a series of novel multi-target directed ligands (MTDLs), i.e., compounds **4a**–**l**, via a convenient one-pot three-component Hantzsch reaction. This approach targeted calcium channel antagonism, antioxidant capacity, cathepsin S inhibition, and interference with Nrf2 transcriptional activation. Of these MTDLs, **4i** emerged as a promising compound, demonstrating robust antioxidant activity, the ability to activate Nrf2-ARE pathways, as well as calcium channel blockade and cathepsin S inhibition. Dihydropyridine **4i** represents the first example of an MTDL that combines these biological activities.

## 1. Introduction

Alzheimer’s disease (AD) is a complex neurodegenerative disease of the central nervous system, characterized by symptoms like memory loss, cognitive impairment and behavioural changes. It primarily affects the elderly population [1]. The number of Alzheimer’s patients is expected to reach 115 million by 2050, with significant social and economic consequences [2]. Despite its substantial impact, effective pharmaceutical solution for the disease remain limited to date [3]. Existing treatments offer only temporary relief of symptoms, and innovative monoclonal antibodies do not provide a definitive cure either.

AD possesses a multifactorial pathogenesis characterized by the accumulation of amyloid beta peptide (Aβ) and the formation of neurofibrillary tangles composed of hyperphosphorylated tau protein [4]. These pathological structures lead to the gradual loss of cholinergic neurons and low levels of acetylcholine, resulting in memory loss and cognitive dysfunction [5].

Elevated intracellular calcium levels play a central role in AD progression. Calcium entry through L-type Ca^2+^ channels disrupts mitochondria and leads to cell death. High calcium levels also promote the formation of Aβ [6,7] and tau pathology [8]. This complex interplay between calcium, Aβ, and tau protein highlights the etiology of AD, and strategies that disrupt this cycle, such as voltage-gated calcium channel (VGCC) blockers, hold promise as a potential therapy.

Cathepsin S (Cat S), a lysosomal cysteine protease enzyme, plays a critical role in protein and antigen degradation within lysosomes. Cat S overexpression is observed in individuals with Down syndrome (DS), who also show Aβ pathology in hippocampal and neocortical neurons in the temporal lobe [9,10], and is associated with AD [9] as it facilitates tau aggregation in vitro [11]. Hence, selective cathepsin S inhibitors are emerging as potential therapeutics for the treatment of these neurodegenerative diseases [10].

Oxidative stress (OS) is another critical factor in the AD pathogenesis, resulting from a combination of events, including mitochondrial dysfunction, biometal imbalance, neuroinflammation, and hydrogen peroxide production [12,13,14,15]. OS is the result of an imbalance between several endogenous antioxidant/pro-oxidant mechanisms, resulting in the oxidant species overproduction. The Keap1-Nrf2-ARE signaling pathway regulates antioxidant defenses and plays a crucial role in the OS and detoxification processes when activated. This pathway involves several key components: Keap1 (Kelch-like ECH-associated protein 1), Nrf2 (nuclear factor (erythroid-derived 2)-like 2), and ARE (antioxidant response element) [16].

In light of the above, the development of multitarget directed ligands (MTDLs), i.e., single small molecules that modulate multiple biological targets responsible for AD pathogenesis, has been the focus of intense research [17,18,19,20,21].

**Figure 1:**
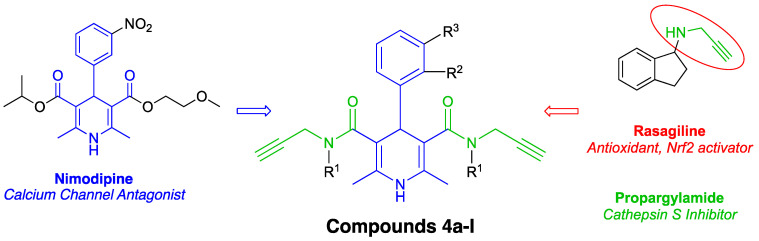
Design of compounds **4a**–**l**.

Our research focuses on multicomponent reactions (MCRs), not only for their ability to generate high structural diversity but also as environmentally friendly synthesis tools [22,23,24]. Continuing our contributions in this field, we present the design, synthesis through Hantzsch-MCR, and the biological assessment of a novel family of MTDLs (**4a**–**l**). These compounds have been designed (see Figure 1) based on the combination of dihydropyridines (DHPs), which exhibit an inherent calcium channel modulation activity [25,26], and propargyl amide residues as electrophilic substructures.

DHPs play a crucial role in medicinal chemistry, serving as the fundamental building blocks for widely used calcium channel antagonists such as nifedipine, nivaldipine, and others. Importantly, elevated cytosolic calcium levels, implicated in the pathogenesis of AD, contribute to the generation of Aβ peptides through calcium-mediated β-secretase activity [6,7,27,28,29]. They also regulate glycogen synthase kinase, leading to tau hyperphosphorylation and increased neurofibrillary tangle (NFT) formation [8]. Consequently, calcium channel antagonists, including DHPs, may exert a neuroprotective influence by inhibiting the development of Aβ peptides and NFTs, which are key features of AD [30].

Propargylamines are known as antioxidant residues capable of effectively scavenging reactive oxygen species (ROS) and reactive nitrogen species (RNS) [31,32,33]. The propargyl moiety in trade drugs, such as selegiline and rasagiline, is responsible for the nuclear translocation of Nrf2 activity and is able to enhance its binding to the ARE [34]. Additionally, propargyl amides have been studied as irreversible inhibitors of cysteine proteases such as Cap S [35]. Thus, including this feature in the structure of these hybrid compounds could improve their pharmacological profile, providing an additional neuroprotective effect [36].

## 2. Materials and Methods

Melting points (°C) were determined using a Kofler hot bench and are reported without correction.

The reaction progress was monitored using thin-layer chromatography (TLC, Silica gel 60 F254, 0.25 mm thickness; Merck, Darmstadt, Germany). NMR spectra were recorded on a Bruker Avance DRX 400 instrument in either CDCl3 containing tetramethylsilane or DMSO-*d*_6_ as solvents. ^1^H and ^13^C chemical shifts are reported in part per million (ppm) using tetramethylsilane (0.00 ppm) or residual solvent (DMSO-*d*_6_, 2.50/39.52 ppm) as an internal reference standard. BRUKER micro TOF-Q mass spectrometers were used to acquire the mass spectra at UCA Clermont Ferrand, France. The elemental analyses were carried out on a Carlo Erba EA 1108 analyser

**General procedure for the synthesis of acetoacetamides (2a**–**b).** Acetoacetamides **(2a**–**b)** were synthesized as described by Clemens et al. [37] A solution of 2,2,6-trimethyl-4*H*-1,3-dioxin-4-one (1 equiv.) and the corresponding propargylic amine (1 equiv.) was refluxed in xylene (10 mmol/mL) over 150 min. The reaction was quenched by adding CH_2_Cl_2,_ and solvents were removed in vacuo. The residue was purified by flash column to afford final acetoacetamides with 25–72% of yields.

**3-Oxo-*N*-(prop-2-yn-1-yl)butanamide (2a).** The crude material was synthesized following the general procedure from propargylamine **1a** (1 equiv., 50 mmol, 6.67 mL) and 2,2,6-trimethyl-4*H*-1,3-dioxin-4-one (1 equiv., 50 mmol, 3.20 mL) in xylene (5 mL) to afford 3.50 g of the desired compound (25%). ^1^H NMR (400 MHz, CDCl_3_) δ 7.33 (s, 1H), 4.06 (dd, *J* = 5.2, 2.5 Hz, 2H), 3.44 (s, 2H), 2.26 (s, 3H), 2.22 (t, *J* = 2.4 Hz, 1H), 1.24 (s, 1H). ^13^C NMR (101 MHz, CDCl_3_) δ 204.51, 165.40, 79.30, 71.71, 49.16, 31.20, 29.20.

***N*-Methyl-3-oxo-*N*-(prop-2-yn-1-yl)butanamide (2b).** The crude material was synthesized following the general procedure from *N*-methylpropargylamine **1b** (1 equiv., 22.58 mmol, 1.90 mL) and 2,2,6-trimethyl-4*H*-1,3-dioxin-4-one (1 equiv., 22.85 mmol, 3 mL) in xylene (6 mL) to afford 2.5 g of the desired compound (72%). ^1^H NMR (400 MHz, CDCl_3_) δ 4.17 (t, *J* = 2.4 Hz, 1.30H) and 3.97 (t, *J* = 2.1 Hz, 0.70H), 3.55 (d, *J* = 1.9 Hz, 0.60H) and 3.49 (d, *J* = 1.9 Hz, 1.40H), 2.99 (d, *J* = 2.2 Hz, 1.27H) and 2.95 (d, *J* = 2.9 Hz, 1.73H), 2.31 (t, *J* = 2.5 Hz, 0.25H) and 1.90 (t, *J* = 2.3 Hz, 0.75H), 2.24–2.16 (m, 2H). ^13^C NMR (101 MHz, CDCl_3_) δ 202.28 and 202.00, 175.53 and 166.49, 78.18, 73.35 and 72.21, 49.92 and 49.80, 40.01, and 36.27, 34.98 and 33.47, 30.33 and 30.21.

**General procedure for the synthesis of Hantzsch derivatives (4a**–**l).** Commercial aldehyde (1 equiv.) and corresponding acetoacetamide (2 equiv.) were dissolved in a minimum volume of EtOH. Subsequently, an equivalent volume of water was gradually added drop by drop, accompanied by ultrasonic treatment of the crude mixture. Ammonium carbonate (1.2 equiv.) was finally added, and the reaction was stirred and heated overnight. The desired compounds (**4a**–**l**) are obtained by filtration, recrystallization, or purification by flash column chromatography, affording yields from 5 to 55%.

**2,6-Dimethyl-4-phenyl-*N*3*,N*5-bis(prop-2-yn-1-yl)-1,4-dihydropyridine-3,5-dicarboxamide (4a).** This compound was prepared according to the general procedure from benzaldehyde (1 equiv., 1.79 mmol, 0.18 mL), 3-oxo-*N*-(prop-2-yn-1-yl)butanamide **2a** (2 equiv., 3.59 mmol, 500 mg), and ammonium carbonate (1.2 equiv., 2.15 mmol, 207 mg) at 35 °C over 14 h. The precipitated **4a** was filtered, triturated in a mixture of pentane and diethyl ether (1:1 *v*/*v*), and then filtered again, ultimately yielding 48.20 mg (15%) as a beige powder. ^1^H NMR (400 MHz, DMSO-*d*_6_) δ 7.96 (s, 1H), 7.72 (t, *J* = 5.5 Hz, 2H), 7.21–7.05 (m, 5H), 4.75 (s, 1H), 3.85 (qdd, *J* = 17.4, 5.5, 2.4 Hz, 4H), 3.04 (t, *J* = 2.4 Hz, 2H), 2.08 (s, 6H). ^13^C NMR (101 MHz, DMSO-*d*_6_) δ 168.05, 146.63, 138.86, 128.02, 126.91, 125.86, 104.04, 81.80, 72.44, 27.86, 17.35. Anal. Calcd. for C_21_H_20_N_3_O_2_: C, 72.60; H, 6.09; N, 12.10; found: C, 70.86; H, 6.19; N, 12.23.

**2,6-Dimethyl-4-(3-nitrophenyl)-*N3,N5*-bis(prop-2-yn-1-yl)-1,4-dihydropyridine-3,5-dicarboxamide (4b). Compound 4b** was prepared according to the general procedure starting from 2-nitrobenzaldehyde (1 equiv., 1.79 mmol, 270 mg), 3-oxo-*N*-(prop-2-yn-1-yl)butanamide **2a** (2 equiv., 3.59 mmol, 500 mg), and ammonium carbonate (2.3 equiv., 4.32 mmol, 414 mg). After 15 h, H_2_O was poured into the reaction crude, extracted 5 times with CH_2_Cl_2_, and then dried over Na_2_SO_4_. The substance was filtered and subjected to purification through flash column chromatography using CH2Cl2/MeOH (95/5) with 1% NH3 as the eluent. This process resulted in the final yield of 121 mg (8.75%) of 4b in the form of bright orange crystals. ^1^H NMR (400 MHz, DMSO-*d*_6_) δ 8.16 (s, 1H), 7.70–7.57 (m, 4H), 7.51 (dd, *J* = 7.9, 1.4 Hz, 1H), 7.38–7.29 (m, 1H), 5.25 (s, 1H), 3.77 (dddd, *J* = 51.5, 17.4, 5.4, 2.5 Hz, 4H), 3.01 (t, *J* = 2.5 Hz, 2H), 2.06 (s, 6H). ^13^C NMR (101 MHz, DMSO-*d*_6_) δ 167.43, 147.08, 141.59, 139.14, 133.44, 130.96, 127.27, 123.29, 104.08, 81.27, 72.70, 36.06, 27.96, 17.39. Anal. Calcd. for C_21_H_20_N_4_O_4_: C, 64.28; H, 5.14; N, 14.28; found: C, 63.11; H, 5.19; N, 14.39.

**2,6-Dimethyl-4-(2-nitrophenyl)-*N3*,*N5*-bis(prop-2-yn-1-yl)-1,4-dihydropyridine-3,5-dicarboxamide (4c).** This compound was prepared according to the general procedure from 3-nitrobenzaldehyde (1 equiv., 1.79 mmol, 272 mg), 3-oxo-*N*-(prop-2-yn-1-yl)butanamide **2a** (2 equiv., 3.59 mmol, 500 mg), and ammonium carbonate (1.2 equiv., 2.16 mmol, 207 mg) at 50 °C over 15 h. Precipitated **4c** was filtered, washed in diethyl ether, and filtered again to finally afford 275.5 mg (39%) as a bright yellow powder. ^1^H NMR (400 MHz, DMSO-*d*_6_) δ 8.13 (s, 1H), 8.01–7.93 (m, 2H), 7.89 (t, *J* = 5.6 Hz, 2H), 7.59 (d, *J* = 7.7 Hz, 1H), 7.50 (td, *J* = 7.7, 1.2 Hz, 1H), 4.92 (s, 1H), 3.89–3.74 (m, 4H), 2.98 (t, *J* = 2.4 Hz, 2H), 2.08 (s, 6H). ^13^C NMR (101 MHz, DMSO-*d*_6_) δ 167.67, 148.96, 147.71, 139.11, 133.94, 129.45, 121.68, 120.99, 103.62, 81.69, 72.28, 40.52, 27.88, 17.38. Anal. Calcd. for C_21_H_20_N_4_O_4_: C, 64.28; H, 5.14; N, 14.28; found: C, 63.39; H, 5.11; N, 14.39.

**4-(2,1,3-Benzoxadiazol-4-yl)-2,6-dimethyl-*N3,N5*-bis(prop-2-yn-1-yl)-1,4-dihydropyridine-3,5-dicarboxamide (4d).** Compound **4d** was prepared according to the general procedure from 2,1,3-benzoxadiazole-4-benzaldehyde (1 equiv., 0.72 mmol, 107 mg), 3-oxo-*N*-(prop-2-yn-1-yl)butanamide 2a (2 equiv., 1.47 mmol, 200 mg) and ammonium carbonate (1.2 equiv., 0.86 mmol, 83 mg) at 50 °C over 15 h. Then, the mixture was cooled to room temperature, and precipitated 4d was filtered and resuspended in toluene. The solvent was removed under reduced pressure, and the residue was washed with diethyl ether and filtered again to finally afford 141.5 mg (51%) of 4d as a dark yellowish powder. ^1^H NMR (400 MHz, DMSO-*d*_6_) δ 7 δ 8.15 (s, 1H), 7.87–7.73 (m, 3H), 7.46 (dd, *J* = 8.8, 6.8 Hz, 1H), 7.13 (d, *J* = 6.6 Hz, 1H), 5.23 (s, 1H), 3.90–3.67 (m, 4H), 2.96 (s, 2H), 2.03 (s, 6H). ^13^C NMR (101 MHz, DMSO-*d*_6_) δ 167.78, 149.51, 148.05, 138.95, 134.57, 132.92, 127.93, 113.41, 102.32, 81.64, 72.29, 27.88, 17.27. Anal. Calcd. for C_21_H_19_N_5_O_5_: C, 64.77; H, 4.92; N, 17.98; found: C, 62.89; H, 4.88; N, 18.08.

**4-(2-Chlorophenyl)-2,6-dimethyl-*N3,N5*-bis(prop-2-yn-1-yl)-1,4-dihydropyridine-3,5-dicarboxamide (4e).** This compound was prepared according to the general procedure starting from 2-chlorobenzaldehyde (1 equiv., 1.80 mmol, 0.20 mL), 3-oxo-*N*-(prop-2-yn-1-yl)butanamide 2a (2 equiv., 3.59 mmol, 500 mg), and ammonium carbonate (1.2 equiv., 2.16 mmol, 207 mg) at 45 °C over 15 h. Then, it was cooled to room temperature and extracted with CH_2_Cl_2_ three times. Organic layers were combined, dried over Na_2_SO_4,_ and evaporated. The residue was washed with diethyl ether and filtered to afford 129 mg (56%) of 4e as a white powder. ^1^H NMR (400 MHz, DMSO-*d*_6_) δ 7.96 (s, 1H), 7.72 (s, 2H), 7.28 (d, *J* = 7.3 Hz, 1H), 7.24–7.19 (m, 2H), 7.10 (t, *J* = 7.1 Hz, 1H), 5.10 (s, 1H), 3.82 (d, *J* = 2.7 Hz, 4H), 3.04 (s, 2H), 2.01 (s, 6H). ^13^C NMR (101 MHz, DMSO-*d*_6_) δ 167.98, 144.83, 137.89, 130.79, 130.16, 128.73, 127.61, 127.48, 104.47, 81.49, 72.69, 40.20, 27.89, 16.97. Anal. Calcd. for C_21_H_20_ClN_3_O_2_: C, 66.05; H, 5.28; N, 11.00; found: C, 64.86; H, 5.31; N, 10.91.

**4-(2-Methoxyphenyl)-2,6-dimethyl-*N3,N5*-bis(prop-2-yn-1-yl)-1,4-dihydropyridine-3,5-dicarboxamide (4f).** Compound **4f** was prepared according to the general procedure starting from 2-methoxybenzaldehyde (1 equiv., 0.72 mmol, 0.087 mL), 3-oxo-N-(prop-2-yn-1-yl)butanamide **2a** (2 equiv., 1.44 mmol, 200 mg), and ammonium carbonate (1.7 equiv., 0.86 mmol, 119 mg) at 50 °C over 15 h. Then, it was cooled to room temperature, and water was poured into the mixture. Precipitated **4f** was filtered and washed with diethyl ether to afford 78.7 mg (30%) as a light yellowish solid. ^1^H NMR (400 MHz, DMSO-*d*_6_) δ 8.05 (s, 1H), 7.42 (s, 2H), 7.20–7.05 (m, *J* = 16.1, 7.6 Hz, 3H), 6.86 (t, *J* = 8.2 Hz, 2H), 4.92 (s, 1H), 3.92–3.74 (m, 7H), 3.07 (s, 2H), 2.12 (s, 6H). ^13^C NMR (101 MHz, DMSO-*d*_6_) δ 167.47, 153.60, 139.92, 135.88, 129.43, 127.43, 121.29, 110.45, 104.13, 81.50, 72.80, 55.56, 32.69, 28.11, 17.55. HRMS ESI-TOF [M]^+^ *m/z* calcd. for C_22_H_23_N_3_O_3_: 377,1724, found: 377,1739.

**4-(2-Bromophenyl)-2,6-dimethyl-*N3,N5*-bis(prop-2-yn-1-yl)-1,4-dihydropyridine-3,5-dicarboxamide (4g).** This compound was prepared according to the general procedure starting from 2-bromobenzaldehyde (1 equiv., 1.08 mmol, 0.126 mL), 3-oxo-*N*-(prop-2-yn-1-yl)butanamide **2a** (2 equiv., 2.16 mmol, 300 mg), and ammonium carbonate (1.2 equiv., 1.29 mmol, 124 mg) at 45 °C over 15 h. Precipitated 4g was filtered and washed with diethyl ether over 1 h to afford 154 mg (34%) as a white solid. ^1^H NMR (400 MHz, DMSO-*d*_6_) δ 7.96 (s, 1H), 7.71 (t, *J* = 5.1 Hz, 2H), 7.38 (d, *J* = 7.9 Hz, 1H), 7.32–7.19 (m, 2H), 7.09–6.96 (m, 1H), 5.07 (s, 1H), 3.95–3.75 (m, 4H), 3.05 (s, 2H), 2.00 (s, 6H). ^13^C NMR (101 MHz, DMSO-*d*_6_) δ 167.98, 146.90, 137.54, 132.00, 131.03, 128.18, 128.00, 120.49, 104.90, 81.47, 72.78, 41.59, 27.89, 16.91. Anal. Calcd. for C_22_H_23_BrN_3_O_3_: C, 59.17; H, 4.73; N, 9.86; found: C, 58.79; H, 4.80; N, 9.88.

**4-(3-Bromophenyl)-2,6-dimethyl-*N3,N5*-bis(prop-2-yn-1-yl)-1,4-dihydropyridine-3,5-dicarboxamide (4h).** Compound **4h** was prepared according to the general procedure starting from 2-bromobenzaldehyde (1 equiv., 1.08 mmol, 0.126 mL), 3-oxo-N-(prop-2-yn-1-yl)butanamide **2a** (2 equiv., 2.157 mmol, 300 mg), and ammonium carbonate (1.2 equiv., 1.29 mmol, 124 mg) at 45 °C over 15 h. Precipitated **4h** was filtered and washed with diethyl ether over 1h to afford 56.8 mg (12%) as a white solid. ^1^H NMR (400 MHz, DMSO-*d*_6_) δ 8.03 (s, 1H), 7.80 (t, *J* = 5.1 Hz, 2H), 7.33–7.25 (m, 2H), 7.20–7.07 (m, 2H), 4.77 (s, 1H), 3.84 (qd, *J* = 17.3, 3.1 Hz, 4H), 3.02 (s, 2H), 2.07 (s, 6H). ^13^C NMR (101 MHz, DMSO-*d*_6_) δ 167.82, 149.33, 139.00, 130.21, 129.64, 128.76, 126.03, 121.56, 103.68, 81.74, 72.42, 40.34, 27.87, 17.36. Anal. Calcd. for C_22_H_23_BrN_3_O_3_: C, 59.17; H, 4.73; N, 9.86; found: C, 57.88; H, 4.69; N, 9.78.

**4-(2-Chlorophenyl)-*N*2,*N*3,5,6-tetramethyl-*N*3,*N*5-bis(prop-2-yn-1-yl)-1,4-dihydropyridine-3,5-dicarboxamide (4i).** This compound was prepared according to the general procedure from 2-chlorobenzaldehyde (1 equiv., 1.09 mmol, 0.126 mL), *N*-methyl-3-oxo-*N*-(prop-2-yn-1-yl)butanamide **2b** (3 equiv., 3.27 mmol, 500 mg), and ammonium carbonate (1.2 equiv., 1.96 mmol, 188 mg) at 55 °C over 19 h. CH_2_Cl_2_ was added to the mixture. The organic layer was washed with water and brine, then dried over Na_2_SO_4_ and evaporated to finally be purified by flash column chromatography using 25/75 hexane/EtOAc + 1% Et_3_N as eluent. 4i was washed with pentane:diethyl ether 1:1 *v/v* and filtered to finally afford 95 mg (12%) as a light beige solid. ^1^H NMR (400 MHz, DMSO-*d*_6_) δ 7.78 (s, 1H), 7.38 (bs, 1H), 7.29–7.19 (m, 2H), 7.16–7.07 (m, 1H), 4.93 (s, 1H), 4.00 (t, *J* = 19.3 Hz, 4H), 3.13 (s, 2H), 2.78 (s, 6H), 1.74 (s, 6H). ^13^C NMR (101 MHz, DMSO-*d*_6_) δ 169.92, 133.23, 131.20, 127.80, 127.32, 79.15, 74.33, 15.94. Anal. Calcd. for C_23_H_24_ClN_3_O_2_: C, 67.39; H, 5.90; N, 10.25; found: C, 66.12; H, 5.98; N, 10.30.

**4-(2,3-Dichlorophenyl)-*N*2,*N*3,5,6-tetramethyl-*N*3,*N*5-bis(prop-2-yn-1-yl)-1,4-dihydropyridine-3,5-dicarboxamide (4j).** Compound **4j** was prepared according to the general procedure starting from 2,3-dichlorobenzaldehyde (1 equiv., 1.14 mmol, 200 mg), *N*-methyl-3-oxo-*N*-(prop-2-yn-1-yl)butanamide 2b (2 equiv., 2.29 mmol, 350 mg), and ammonium carbonate (1.2 equiv., 1.37 mmol, 140 mg) at 45 °C over 19 h. Diethyl ether was added to the reaction crude. The organic layer was washed with water and brine, then dried over Na_2_SO_4_, and evaporated under reduced pressure to finally be purified by flash column chromatography using 94/6 CH_2_Cl_2_/MeOH + 1% NH_3_ as eluent. 4j was washed with pentane:diethyl ether 1:1 *v/v* and filtered to finally afford 21.3 mg (7%) as a light yellow powder. ^1^H NMR (400 MHz, DMSO-*d*_6_) δ 7.88 (s, 1H), 7.44–7.34 (m, 2H), 7.30 (d, *J* = 6.7 Hz, 1H), 4.99 (s, 1H), 4.05 (dd, *J* = 30.9, 17.2 Hz, 4H), 3.13 (s, 2H), 2.81 (s, 6H), 1.74 (s, 6H). ^13^C NMR (101 MHz, DMSO-*d*_6_) δ 169.72, 133.66, 129.72, 128.33, 128.11, 101.86, 74.33, 15.95, 13.90. HRMS ESI-TOF [M]^+^ m/z calcd. for C_23_H_23_Cl_2_N_3_O_2_: 443,1149, found: 443,1167.

**4-(2-Methoxyphenyl)-*N*2,*N*3,5,6-tetramethyl-*N*3,*N*5-bis(prop-2-yn-1-yl)-1,4-dihydropyridine-3,5-dicarboxamide (4k).** This compound was prepared according to the general procedure starting from 2-methoxybenzaldehyde (1 equiv., 1.63 mmol, 0.197 mL), *N*-methyl-3-oxo-*N*-(prop-2-yn-1-yl)butanamide **2b** (2 equiv., 3.27 mmol, 500 mg), and ammonium carbonate (1.2 equiv., 1.96 mmol, 188 mg) at 40 °C over 15 h. Diethyl ether was added to the reaction crude. The organic layer was washed with water and brine, then dried over Na2SO4 and reduced under pressure conditions to finally be purified by flash column chromatography using 96/4 CH_2_Cl_2_/MeOH + 1% NH_3_ as eluent. 4k was washed with pentane:diethyl ether 1:1 *v/v* over 1 h and filtered to finally afford 45.6 mg (7%) as a light beige powder. ^1^H NMR (400 MHz, DMSO-*d*_6_) δ 7.60 (s, 1H), 7.18–7.04 (m, 2H), 6.83 (dd, *J* = 16.3, 7.9 Hz, 2H), 4.78 (s, 1H), 4.01 (t, *J* = 20.9 Hz, 4H), 3.62 (s, 3H), 3.15 (s, 2H), 2.76 (s, 6H), 1.73 (s, 6H). ^13^C NMR (101 MHz, DMSO-*d*_6_) δ 170.58, 129.27, 127.22, 120.25, 110.47, 102.15, 94.29, 79.49, 74.06, 64.90, 55.20, 46.98, 34.46, 15.93. HRMS ESI-TOF [M]^+^ m/z calcd. for C_24_H_27_N_3_O_3_: 405,2042, found: 405,2052.

**4-(3-Methoxyphenyl)-*N2,N3*,5,6-tetramethyl-*N3,N5*-bis(prop-2-yn-1-yl)-1,4-dihydropyridine-3,5-dicarboxamide (4l).** Compound **4l** was prepared according to the general procedure starting from 3-methoxybenzaldehyde (1 equiv., 1.63 mmol, 0.197 mL), *N*-methyl-3-oxo-*N*-(prop-2-yn-1-yl)butanamide 2b (2 equiv., 3.27 mmol, 500 mg), and ammonium carbonate (1.2 equiv., 1.96 mmol, 188 mg) at 45 °C over 15 h. CH_2_Cl_2_ was added to the reaction mixture. The organic layer was washed with water, brine, then dried over Na_2_SO_4_ and evaporated to finally be purified by flash column chromatography using 96/4 CH_2_Cl_2_/MeOH + 1% NH_3_ as eluent. **4l** was washed with pentane:diethyl ether 1:1 *v/v* over 1 h and filtered to finally afford 56.5 mg (5%) as light beige powder. ^1^H NMR (400 MHz, DMSO-*d*_6_) δ δ 7.66 (s, 1H), 7.12 (t, *J* = 7.6 Hz, 1H), 6.67 (dd, *J* = 24.2, 7.4 Hz, 2H), 6.55 (s, 1H), 4.44 (s, 1H), 4.00 (s, 4H), 3.67 (s, 3H), 3.14 (s, 2H), 2.72 (s, 6H), 1.73 (s, 6H). ^13^C NMR (101 MHz, DMSO-*d*_6_) δ 170.48, 159.14, 146.53, 129.15, 119.76, 113.00, 111.48, 102.62, 79.26, 54.84, 44.85, 16.01. HRMS ESI-TOF [M]^+^ m/z calcd. for C_24_H_27_N_3_O_3_: 405.2040, found: 405.2052.

**Calcium channel blockade.** Evaluation of the calcium channel blockade of compounds **4a**–**l** was performed using the FLIPR Calcium 6 indicator according to a previously established protocol [38]. Briefly, human neuroblastoma cell line SH-SY5Y seeded out on 96-well plate were treated with fluorescent calcium indicator (FLIPR Calcium 6, Molecular Devices). Cells were incubated with indicator for 2 h at 37 °C, facilitating internalization of the indicator. Subsequently, cells were exposed to nimodipine (10 µM, used as reference inhibitor), DMSO (0.1%, used as vehicle control) or our compounds of interest (10 µM) for 10 min also at 37 °C. Following that, cell fluorescence was recorded using a microplate reader (λ_Ex_ = 485 nm; λ_Em_ = 525 nm). Baseline fluorescence was recorded for 5 s. Afterwards, cells were stimulated by the addition of the solution of KCl and CaCl_2_ (90 and 5 mM, respectively) in order to induce the opening of voltage-gated calcium channels. An increase in fluorescence of calcium indicator after inducing calcium flux was recorded for another 30 s. Data were collected from three independent experiments, with eight technical replicates per experiment. Fluorescence intensity values were normalized to the baseline recorded before the induction of calcium flux. The percentage of inhibition was calculated as a reduction of normalized calcium flux in comparison with the DMSO vehicle control. Outliers were detected using the Grubbs test, and any outlying values were excluded from further analysis.

**Oxygen radical absorbance capacity assay.** The evaluation of the antioxidant activity of compounds **4a**–**l** was carried out using the previously established ORAC-FL method [38], and detailed information is provided in the supporting information.

**Nrf2 transcriptional activation potencies.** The assessment of the Nrf2 transcriptional activation potencies of compounds **4a**–**l** was carried out using the previously described method [38], and detailed information is provided in the supporting information.

**Cathepsin assays**. Assays with human cathepsins B, S, L, and K were carried out as described previously [39,40,41]. Stock solutions of substrates and inhibitors were prepared in DMSO. The final DMSO concentration was 2%. All test compounds were investigated in duplicate at a concentration of 50 µM to determine residual activities based on endpoints of substrate consumption after 60 min. *K*_i_ values were obtained in duplicate measurements with six different inhibitor concentrations, and 60-min-progress curves were analyzed by linear regression. IC_50_ values were obtained by non-linear regression using the equation *v*_i_ = *v*_0_/(1 + ([I]/IC_50_), where *v*_i_ is the product formation rate at different inhibitor concentrations, *v*_0_ is the uninhibited product formation rate, [I] is the inhibitor concentration, and IC_50_ is the half-maximal inhibitory concentration. IC_50_ values are transformed to *K*_i_ values using the Cheng-Prusoff equation.

Human isolated cathepsin B (Calbiochem) was assayed spectrophotometrically (Cary 50 Bio, Varian) at 405 nm and 37 °C. Assay buffer was 100 mM sodium phosphate buffer pH 6.0, 100 mM NaCl, 5 mM EDTA, 0.01% Brij 35. For activation, an enzyme stock solution was diluted with assay buffer containing 5 mM DTT and incubated for 30 min at 37 °C. The final concentration of the chromogenic substrate Z-Arg-Arg-pNA was 500 µM (0.45 × *K*m).

Human recombinant cathepsin S (Enzo Life Sciences) was assayed fluorometrically (FLUOstar Optima plate reader, BMG Labtech) at 25 °C with an emission wavelength of 360 nm and an absorption wavelength of 460 nm. The assay buffer was 100 mM sodium phosphate buffer pH 6.0, 100 mM NaCl, 5 mM EDTA, 0.01% Brij 35. For activation, an enzyme stock solution was diluted with assay buffer containing 5 mM DTT and incubated for 60 min at 37 °C. The final concentration of the chromogenic substrate Z-Phe-Arg-AMC was 40 µM (0.74 × *K*_m_).

Human isolated cathepsin L (Enzo Life Sciences) was assayed spectrophotometrically (Cary 50 Bio, Varian) at 405 nm and 37 °C. The assay buffer was 100 mM sodium phosphate buffer pH 6.0, 100 mM NaCl, 5 mM EDTA, and 0.01% Brij 35. For activation, an enzyme stock solution was diluted with assay buffer containing 5 mM DTT and incubated for 30 min at 37 °C. The final concentration of the chromogenic substrate Z-Phe-Arg-pNA was 100 µM (5.88 × *K*_m_).

Human recombinant cathepsin K (Enzo Life Sciences) was assayed fluorometrically (FLUOstar Optima plate reader, BMG Labtech) with an emission wavelength of 360 nm and an absorption wavelength of 460 nm. The assay buffer was 100 mM sodium citrate pH 5.0, 100 mM NaCl, 1 mM EDTA, 0.01% CHAPS. For activation, an enzyme stock solution was diluted with assay buffer containing 5 mM DTT and incubated for 30 min at 37 °C. The final concentration of the chromogenic substrate Z-Leu-Arg-AMC was 40 µM (13.33 × *K*_m_).

## 3. Results and Discussion

### 3.1. Synthesis

**Scheme 1:**
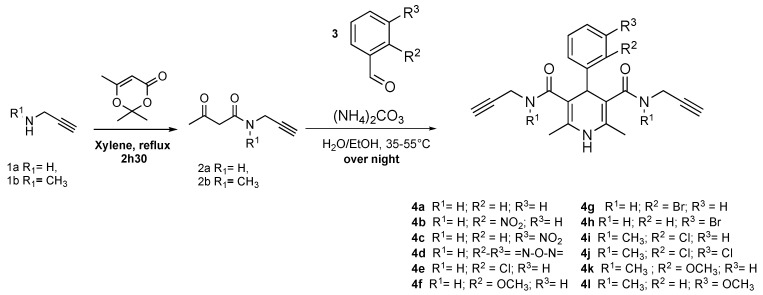
Synthesis of MTDLs **4a**–**l**.

The synthesis of the new MTDL **4a**–**l** was carried out according to the modified Hantzsch reaction protocol reported by Tamaddon et al. [42] using acetoacetamides **2a**–**b**, ammonium carbonate, and aldehydes **3** in a mixture of EtOH/water (1:1 *v*/*v*) at 35–55 °C overnight (Scheme 1). The acetoacetamides were prepared according to the protocol described by Clemens and Hyatt [37]. The reaction started with commercially available prop-2-yn-1-amine (**1a**) or *N*-methylprop-2-yn-1-amine (**1b**) and 2,2,6-trimethyl-4*H*-1,3-dioxin-4-one in xylene at reflux to give the desired acetoacetamide and acetone. All new compounds were characterized using ^1^H and ^13^C NMR and HRMS or elemental analysis, and their structures are collected in the Experimental Section.

### 3.2. Biological Evaluation

**Calcium channel blockade.** The Ca^2+^ channel blocking capacity of compounds **4a**–**l** and nimodipine, used as a standard at a concentration of 10 μM, was evaluated according to an established methodology [38]. As outlined in Table 1, three compounds showed no activity, while the observed percentage values for others ranged from 3 (**4c**) to 19 (**4f**), with nimodipine exhibiting 37%. The most potent compounds, in descending order, were **4f** (19%), **4l** (15%) and **4k** (14%), representing approximately half the activity observed for nimodipine (37%). No structure–activity relationship could be established from these results.

**Antioxidant assay.** The antioxidant assessment was conducted by evaluating the compounds **4a**–**l** through the determination of oxygen radical absorbance capacity (ORAC) using the ORAC-Fluorescein (ORAC-FL) method [43] (Table 1). The compounds’ radical scavenging properties were quantified in Trolox equivalents (TE), using melatonin as a positive control, which exhibited an ORAC value equal to 2.45 [44]. All compounds except **4k** showed antioxidant capacity ranging from 0.86 for **4a** to 1.93 for **4i**. The best-performing compounds, in descending order, were 4i (1.93 TE), 4j (1.82 TE), and 4c (1.54 TE), being, on average, only 1.3 times less active than melatonin. From a structure–activity relationship point of view, the absence of a substituent on the aromatic ring was unfavorable for antioxidant activity. The introduction of electron-withdrawing halogen or nitro groups seems to improve this activity, both for compounds with secondary amide groups (R1 = H; e.g., 4c) and tertiary amide groups (R^1^ = CH_3_, e.g., **4i** and **4j**).

**Nrf2 transcriptional activation potencies of compounds 4a–l.** The Nrf2-ARE activating potential of compounds **4a**–**l** was assessed in vitro by a cell-based luciferase assay using the AREc32 cell line, a reliable cell model for the redox-dependent activation of Nrf2 [45], with melatonin used as a positive control. AREc32 cells were exposed to escalating concentrations of each compound (1, 5, 10, 25, 50, 100, 125, and 150 μM) for 24 h to reach the cytotoxic threshold, followed by measurements of luciferase activity. Melatonin was used as a positive control.

**Figure 2:**
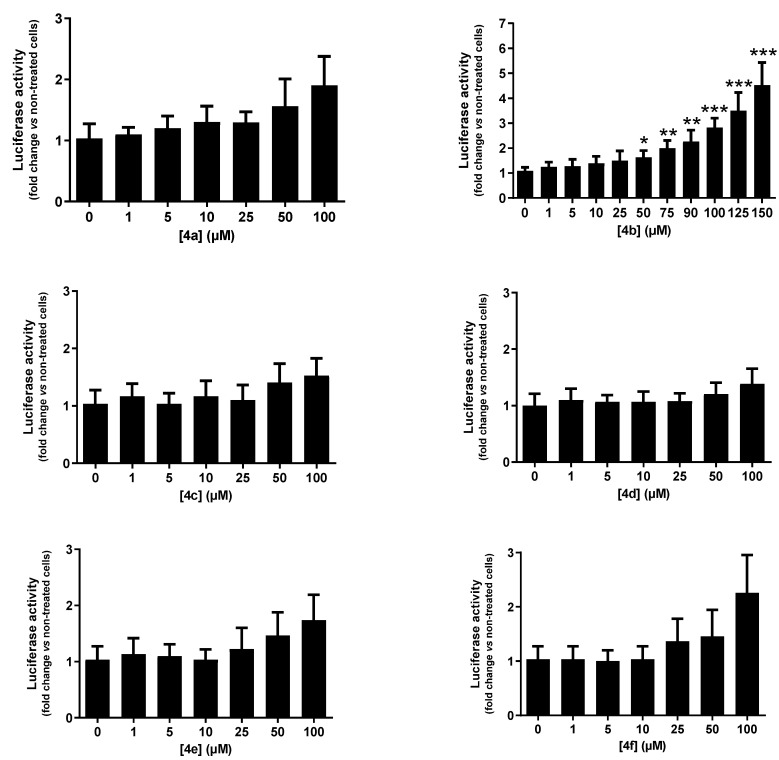
Nrf2 transcriptional activation potencies of compounds **4a**–**f**. Data are means ± SEM of at least 3 different experiments. * *p* ≤ 0.05, ** *p* ≤ 0.01 and *** *p* ≤ 0.001 with respect to non-treated cells (control cells).

**Figure 3:**
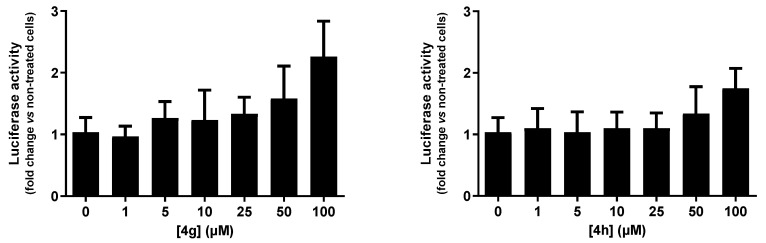

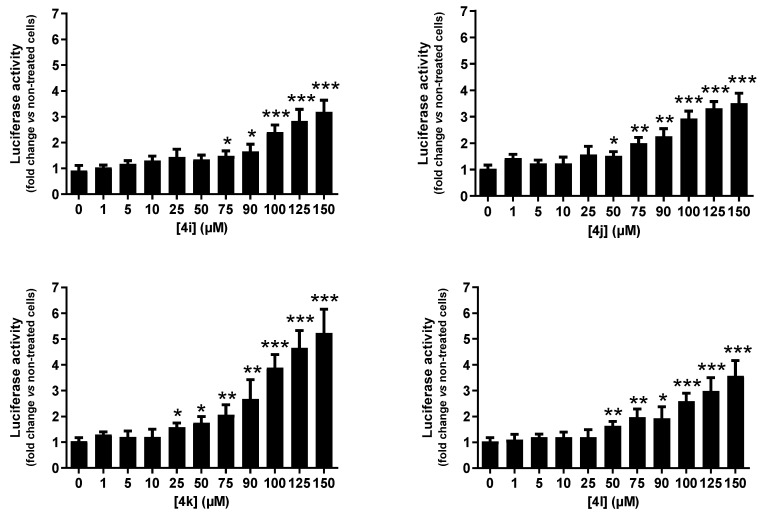
Nrf2 transcriptional activation potencies of compounds **4g**–**l**. Data are means ± SEM of at least 3 different experiments. * *p* ≤ 0.05, ** *p* ≤ 0.01 and *** *p* ≤ 0.001 with respect to non-treated cells (control cells).

In preliminary assessments, the cytotoxicity of the compounds against AREc32 cells was investigated, showing no toxicity of the compounds up to 100 μM. Interestingly, five compounds (**4b**, **4i**–**l**) exhibited no cytotoxicity up to 150 μM, allowing for an investigation of their potential to activate the Nrf2 pathway at this highest concentration. Figure 2 and Figure 3 illustrate that all compounds appear to induce activation of the Nrf2 pathway. However, no significant activity was seen for compounds **4a**, **4c**–**h**. In contrast, **4b** and **4i**–**l** caused a significant induction at concentrations up to 150 μM. In addition, CD values (i.e., the concentration required to double the specific activity of the luciferase reporter) were calculated for these five compounds to assess their relative potencies. As depicted in Table 1, **4b** and **4i**–**l** exhibited CD values of 82.2, 96.0, 77.2, 69.4, and 81.7 μM, respectively, compared to melatonin’s value of 66.4 μM. Notably, **4k** showed comparable activity to melatonin, while **4b**, **4i**, **4j**, and **4l** were only 1.2 to 1.5 times less active than melatonin, which represents a recognized Nrf2 activator capable of inducing transcriptional pathways through various mechanisms [46].

Considering the structure–activity relationship, all compounds with a tertiary amide group (R^1^ = CH_3_) possessed the ability to induce the Nrf2 pathway, with the *ortho*-chloro substitution pattern in **4i** being the most advantageous. Among the primary amide derivatives (R^1^ = H), only the *ortho*-nitro derivative **4b** was active.

**Cathepsins inhibition.** Compounds **4a**–**l** were then evaluated for their ability to inhibit Cat S. Besides Cat S, the set of assays included three related human cathepsins, B, L, and K, all members of the class of lysosomal cysteine proteases (see Table S1 in the Supporting Information). Following reported procedures, [39,40,41] percentage or residual activities were determined using chromogenic or fluorogenic peptide substrates, with endpoints measured after 60 min of substrate consumption. To determine *K*_i_ values, progress curves were followed over 60 min and analyzed by linear regression. *K*_i_ values were then obtained by non-linear regression. Two selective inhibitors of cathepsin S were identified, i.e., **4h** and **4i,** showing *K*_i_ values in the two-digit-micromolar range, 55.3 and 69.3 µM, respectively, see Table 1. Again, no structure–activity relationship could be established from these results. However, when all the results are considered, it is clear that the tertiary amide compounds appear to be more active against all targets than the secondary analogues.

## 4. Conclusions

In this study, we synthesized 12 new compounds via the multi-component Hantzsch reaction, targeting calcium channel inhibition, a strategy whose importance is well established. The design of the compounds was based on the combination of DHPs with propargylamide residues as electrophilic substructures, which proved to be robust and effective. In fact, most of the compounds showed antagonistic activity on calcium channels due to the presence of the DHP scaffold. In addition, almost all the compounds showed antioxidant properties and activated the endogenous Nrf2 antioxidant pathways thanks to the propargylamide moiety. It’s noteworthy that only two compounds were identified as inhibitors of cathepsin S, although propargylamides are known to be studied as irreversible inhibitors of cysteine proteases.

In our biological studies, we identified a promising compound, **4i,** which showed potent antioxidant activity and the ability to activate Nrf2-ARE pathways. In addition, this compound showed a weak inhibitory effect on Cat S and a modest blockade of calcium channels, three times less active than the reference nimodipine.

This study introduced the first generation of MTDLs combining these biological activities and identified **4i** as a promising compound for further research into the treatment of Alzheimer’s disease. Ongoing efforts in our laboratories are aimed at developing analogs with the best pharmacological profile and results will be communicated in due course.

## Figures and Tables

**Table 1:** Biological activities of compounds **4a**–**l**.

Compd	R^1^	R^2^	R^3^	Calcium Channel Blockade (%) ^a^	ORAC ^b^	Nrf2 Induction Potencies CD (µM) ^c^	CatS *K*_i_ (μM) ^d^
**4a**	H	H	H	12	0.86 ± 0.05	n.d. ^e^	n.i. ^f^
**4b**	H	NO_2_	H	n.i.	1.36 ± 0.16	82.8 ± 8.4	n.i.
**4c**	H	H	NO_2_	3	1.54 ± 0.15	n.d.	n.i.
**4d**	H	=N-O-N=		10	1.23 ± 0.23	n.d.	n.i.
**4e**	H	Cl	H	n.i.	1.15 ± 0.02	n.d.	n.i.
**4f**	H	OCH_3_	H	19	1.34 ± 0.20	n.d.	n.i.
**4g**	H	Br	H	n.i.	1.43 ± 0.03	n.d.	n.i.
**4h**	H	H	Br	7	1.28 ± 0.05	n.d.	55.3 ± 7.4
**4i**	CH_3_	Cl	H	11	1.93 ± 0.08	96.0 ± 6.1	69.3 ± 5.2
**4j**	CH_3_	Cl	Cl	12	1.82 ± 0.05	77.2 ± 7.1	n.i.
**4k**	CH_3_	OCH_3_	H	14	n.i.	69.4 ± 9.1	n.i.
**4l**	CH_3_	H	OCH_3_	15	0.96 ± 0.07	81.7 ±11.6	n.i.
nimodipine				37	n.d.	n.d.	n.d.
melatonin				n.d.	2.45 ± 0.09	66.4 ± 16.4	n.d.

^a^ Compounds were tested at a concentration of 10 µM. ^b^ Data are expressed as Trolox equivalents and are the mean (n = 3) ± SEM of two different measurements. ^c^ Nrf2 induction potencies of test compounds in Nrf2/ARE-luciferase reporter cells. ^d^ *K*i (± SEM) values were determined in cases of a residual activity ≤ 60% at 50 μM inhibitor concentration. Measurements were done in duplicates with five different inhibitor concentrations as well as in the absence of inhibitor. ^e^ n.d., not determined, indicates that no experiments were performed. ^f^ n.i., no inhibition, refers to a residual activity ≥ 95% at 50 μM inhibitor concentration.

## Data Availability

Samples of the compounds are available from the authors.

## Supplementary Materials

The following supporting information can be downloaded at https://www.mdpi.com/article/10.3390/pharmaceutics16010121/s1, Table S1: Percentage residual activities of human cathepsins in the presence of inhibitors 4a-l @ 50 µM, Experimental procedure of ORAC test, Experimental procedure of Nrf2 test and NMR Spectras of compounds **4a**–**l**.
