# Supplementary material for: Design and Synthesis of Multi-Functional Ligands through Hantzsch Reaction: Targeting Ca2+ Channels, Activating Nrf2 and Possessing Cathepsin S Inhibitory, and Antioxidant Properties
Source: Pharmaceutics. 2024 Jan 17;16(1):121. doi: 10.3390/pharmaceutics16010121 (PMC10819521; doi:10.3390/pharmaceutics16010121)
Supplement: Supplementary file 1 [file pharmaceutics-16-00121-s001.zip › pharmaceutics-2771084-supplementary.pdf]

Supporting Information

Table of Contents

Table S1..S2

Experimental procedure of ORAC test..S2

Experimental procedure of Nrf2 test...S2

NMR Spectras of compounds 4a-l..S3-S21

Table S1. Percentage residual activities of human cathepsins in the presence of inhibitors **4a-l** @ 50 µM[a]

Compd	R^1	R^2	R^3	Cathepsin			
				B	S	L	K
**4a**	H	H	H	n.i.[b]	68	n.i.	n.i.
**4b**	H	NO_2	H	79	61	n.i.	66
**4c**	H	H	NO_2	n.i.	91	n.i.	72
**4d**	H	=N-O-N=		n.i.	77	90	62
**4e**	H	Cl	H	n.i.	76	n.i.	85
**4f**	H	OCH_3	H	71	67	86	n.i.
**4g**	H	Br	H	88	70	n.i.	90
**4h**	H	H	Br	71	54	87	n.i.
**4i**	CH_3	Cl	H	87	58	91	65
**4j**	CH_3	Cl	Cl	90	69	87	81
**4k**	CH_3	OCH_3	H	86	79	92	82
**4l**	CH_3	H	OCH_3	86	79	88	81

[a] Data from duplicate measurements. Residual enzymatic activity @ 50 µM inhibitor concentration refers to 100% activity in the absence of the inhibitor.
[b] n.i., no inhibition, refers to a residual activity ≥ 95% @ 50 µM inhibitor concentration.

Oxygen radical absorbance capacity assay. The antioxidant activity of **4a**-l was assessed by the ORAC-FL method, using fluorescein as a fluorescent probe. Shortly, fluorescein and the antioxidants were incubated in a black 96-well microplate (Nunc) at 37 °C for 15 min. 2,2'-Azobis(amidinopropane) dihydrochloride was then rapidly added using the built-in injector of a Varioskan Flash plate reader (Thermo Scientific). Fluorescence was measured at 485 nm (excitation wavelength) and 535 nm (emission wavelength) every minute for 1 hour. All reactions were performed in triplicate and a minimum of three different assays were performed for each sample to ensure robustness and reliability of results.

Nrf2 transcriptional activation potencies. The stable NRF2/ARE luciferase reporter HEK293 cell line (Signosis, Santa Clara, CA, USA) was cultured in Dulbecco's MEM high glucose (DMEM) supplemented with 10% FBS and penicillin-streptomycin at 37 °C in a 95% air/5% CO_2 environment. At the start of treatment, cells were seeded at a density of 2×10^{-4} per well in 96-well white microtiter plates. After 48 hours, the culture medium was replaced with fresh DMEM supplemented with 0.1% FBS containing different concentrations of the compounds tested or DMSO (0.1%) in duplicate. Luciferase activity was measured 24 h after treatment using the Bright-Glo luciferase assay system (Promega) according to the manufacturer's instructions. To assess cellular viability, the stable NRF2/ARE luciferase reporter HEK293 cell line was seeded and treated as described for luciferase activity determination, using transparent culture plates instead of white plates. After 24 hours of incubation with the tested compounds, the percentage of cell viability was determined using the MTT assay.

Compound 4a:

2,6-Dimethyl-4-phenyl-*N*3,*N*5-bis(prop-2-yn-1-yl)-1,4-dihydropyridine-3,5-dicarboxamide.

This compound was prepared according to the general procedure from benzaldehyde (1 equiv., 1.79 mmol, 0.18 mL), 3-oxo-*N*-(prop-2-yn-1-yl)butanamide **2a** (2 equiv., 3.59 mmol, 500 mg) and ammonium carbonate (1.2 equiv., 2.15 mmol, 207 mg) at
35 °C over 14 h. The precipitated **4a** was filtered, triturated in a mixture of pentane and diethyl ether (1:1 v/v), and then filtered again, ultimately yielding 48.20 mg (15%) as a beige powder.

^{1}H NMR (400 MHz, DMSO-d_6) δ 7.96 (s, 1H), 7.72 (t, *J* = 5.5 Hz, 2H), 7.21 – 7.05 (m, 5H), 4.75 (s, 1H), 3.85 (qdd, *J* = 17.4, 5.5, 2.4 Hz, 4H), 3.04 (t, *J* = 2.4 Hz, 2H), 2.08 (s, 6H).

^{13}C NMR (101 MHz, DMSO-d_6) δ 168.05, 146.63, 138.86, 128.02, 126.91, 125.86, 104.04, 81.80, 72.44, 27.86, 17.35.

Anal. Calcd. for $C_{21}H_{20}N_3O_2$: C, 72.60; H, 6.09; N, 12.10; found: C, 70.86; H, 6.19; N, 12.23.

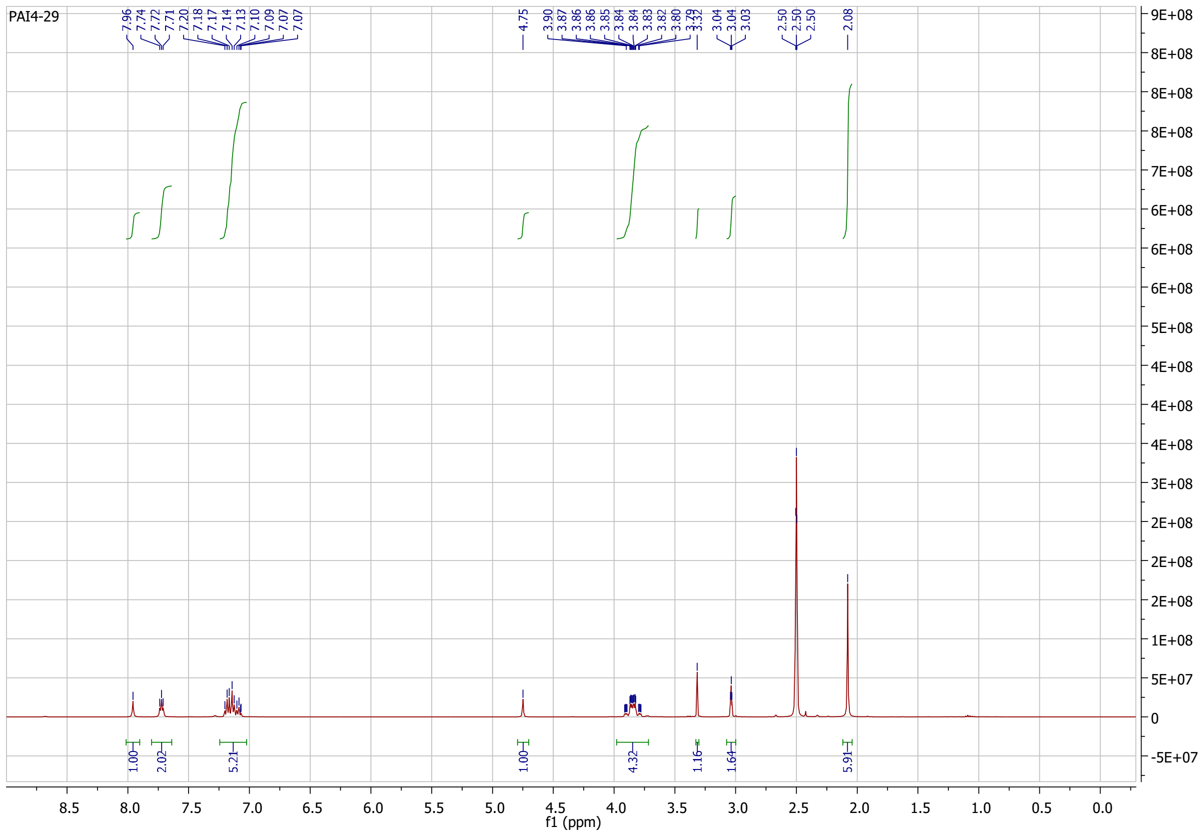

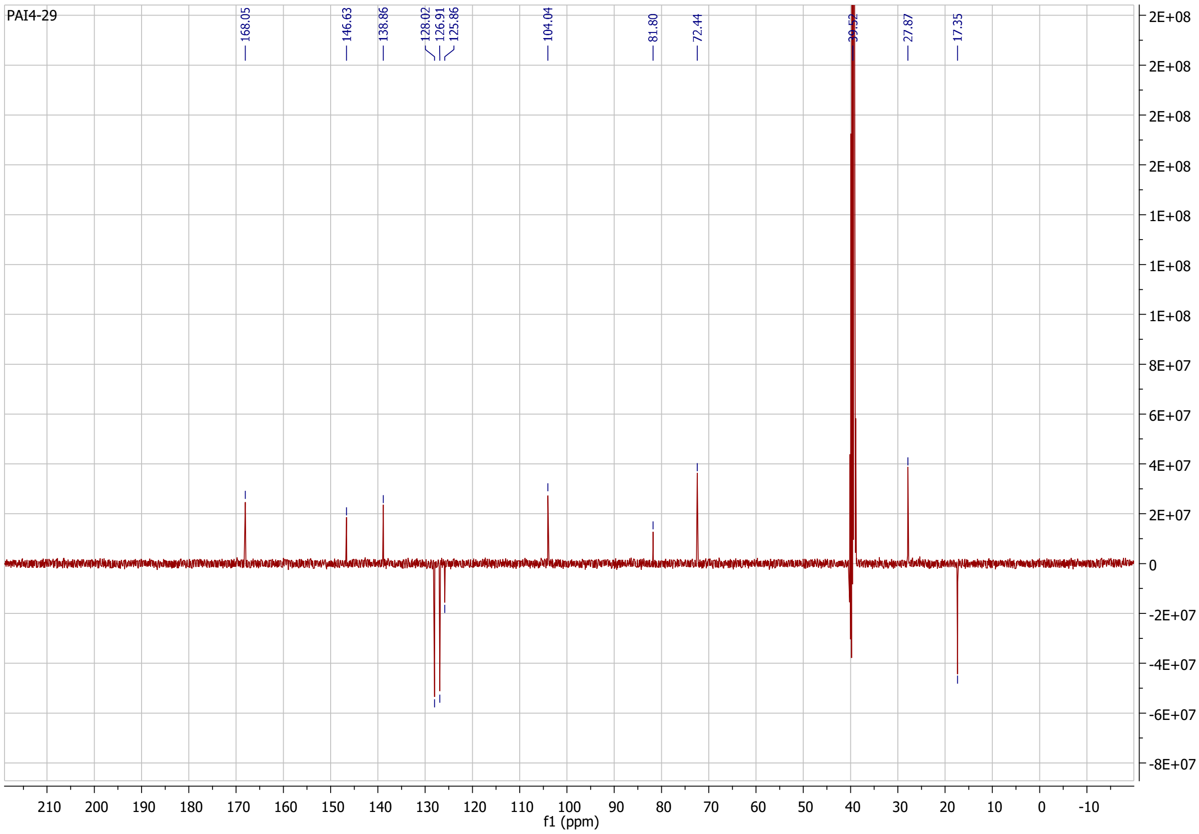

Compound 4b:

2,6-Dimethyl-4-(3-nitrophenyl)-*N3*,*N5*-bis(prop-2-yn-1-yl)-1,4-dihydropyridine-3,5-dicarboxamide.

Compound 4b was prepared according to the general procedure starting from 2-nitrobenzaldehyde (1 equiv., 1.79 mmol, 270 mg), 3-oxo-*N*-(prop-2-yn-1-yl)butanamide **2a** (2 equiv., 3.59 mmol, 500 mg) and ammonium carbonate (2.3 equiv., 4.32 mmol, 414 mg). After 15 h, H_2O was poured into the reaction crude, and extracted 5 times with CH_2Cl_2, then dried over Na_2SO_4. The substance was filtered and subjected to purification through flash column chromatography using CH2Cl2/MeOH (95/5) with 1% NH3 as the eluent. This process resulted in the final yield of 121 mg (8.75%) of 4b in the form of bright orange crystals.

^{1}H NMR (400 MHz, DMSO-d_6) δ 8.16 (s, 1H), 7.70 – 7.57 (m, 4H), 7.51 (dd, *J* = 7.9, 1.4 Hz, 1H), 7.38 – 7.29 (m, 1H), 5.25 (s, 1H), 3.77 (dddd, *J* = 51.5, 17.4, 5.4, 2.5 Hz, 4H), 3.01 (t, *J* = 2.5 Hz, 2H), 2.06 (s, 6H).

^{13}C NMR (101 MHz, DMSO-d_6) δ 167.43, 147.08, 141.59, 139.14, 133.44, 130.96, 127.27, 123.29, 104.08, 81.27, 72.70, 36.06, 27.96, 17.39.

Anal. Calcd. for $C_{21}H_{20}N_4O_4$: C, 64.28; H, 5.14; N, 14.28; found: C, 63.11; H, 5.19; N, 14.39.

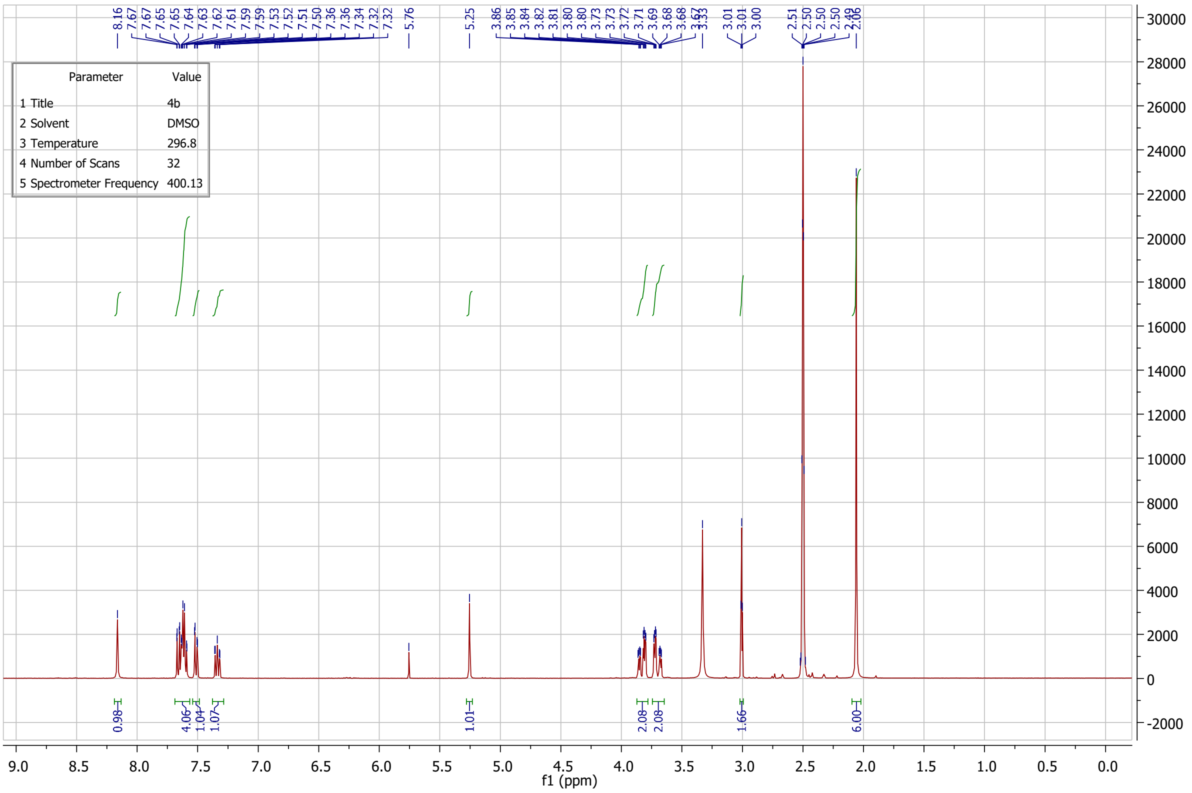

^{1}H NMR (400 MHz, DMSO) δ 8.16 (s, 1H), 7.70 – 7.57 (m, 4H), 7.51 (dd, J = 7.9, 1.4 Hz, 1H), 7.38 – 7.29 (m, 1H), 5.25 (s, 1H), 3.77 (dddd, J = 31.5, 17.4, 5.4, 2.5 Hz, 4H), 3.01 (t, J = 2.5 Hz, 2H), 2.06 (s, 6H).

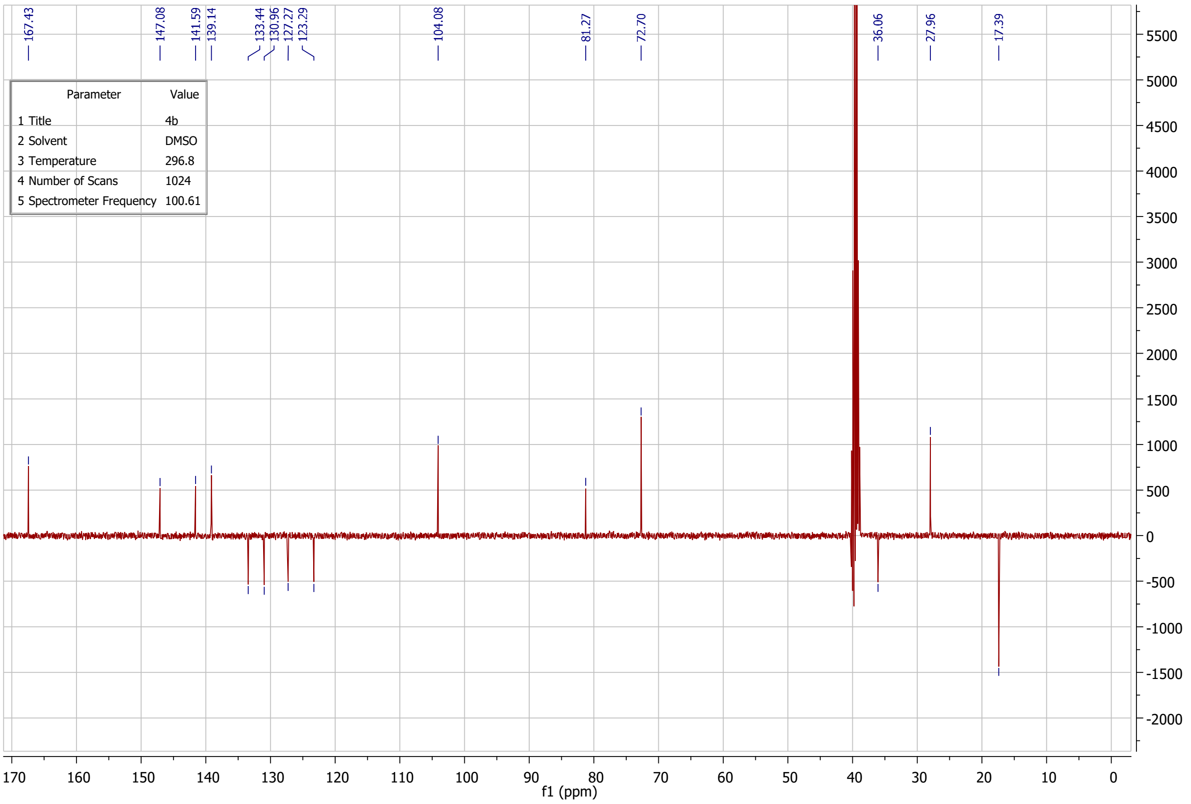

^{13}C NMR (101 MHz, DMSO) δ 167.43, 147.08, 141.59, 139.14, 133.44, 130.96, 127.27, 123.29, 104.08, 81.27, 72.70, 36.06, 27.96, 17.39.

Compound 4c:

2,6-Dimethyl-4-(2-nitrophenyl)-*N3*,*N5*-bis(prop-2-yn-1-yl)-1,4-dihydropyridine-3,5-dicarboxamide.
This compound was prepared according to the general procedure from 3-nitrobenzaldehyde (1 equiv., 1.79 mmol, 272 mg), 3-oxo-*N*-(prop-2-yn-1-yl)butanamide **2a** (2 equiv., 3.59 mmol, 500 mg) and ammonium carbonate (1.2 equiv., 2.16 mmol, 207 mg) at 50 °C over 15 h. Precipitated **4c** was filtered, washed in diethyl ether and filtered again to finally afford 275.5 mg (39%) as bright yellow powder.

^{1}H NMR (400 MHz, DMSO-d_6) δ 8.13 (s, 1H), 8.01 – 7.93 (m, 2H), 7.89 (t, *J* = 5.6 Hz, 2H), 7.59 (d, *J* = 7.7 Hz, 1H), 7.50 (td, *J* = 7.7, 1.2 Hz, 1H), 4.92 (s, 1H), 3.89 – 3.74 (m, 4H), 2.98 (t, *J* = 2.4 Hz, 2H), 2.08 (s, 6H).

^{13}C NMR (101 MHz, DMSO-d_6) δ 167.67, 148.96, 147.71, 139.11, 133.94, 129.45, 121.68, 120.99, 103.62, 81.69, 72.28, 40.52, 27.88, 17.38.

Anal. Calcd. for $C_{21}H_{20}N_4O_4$: C, 64.28; H, 5.14; N, 14.28; found: C, 63.39; H, 5.11; N, 14.39.

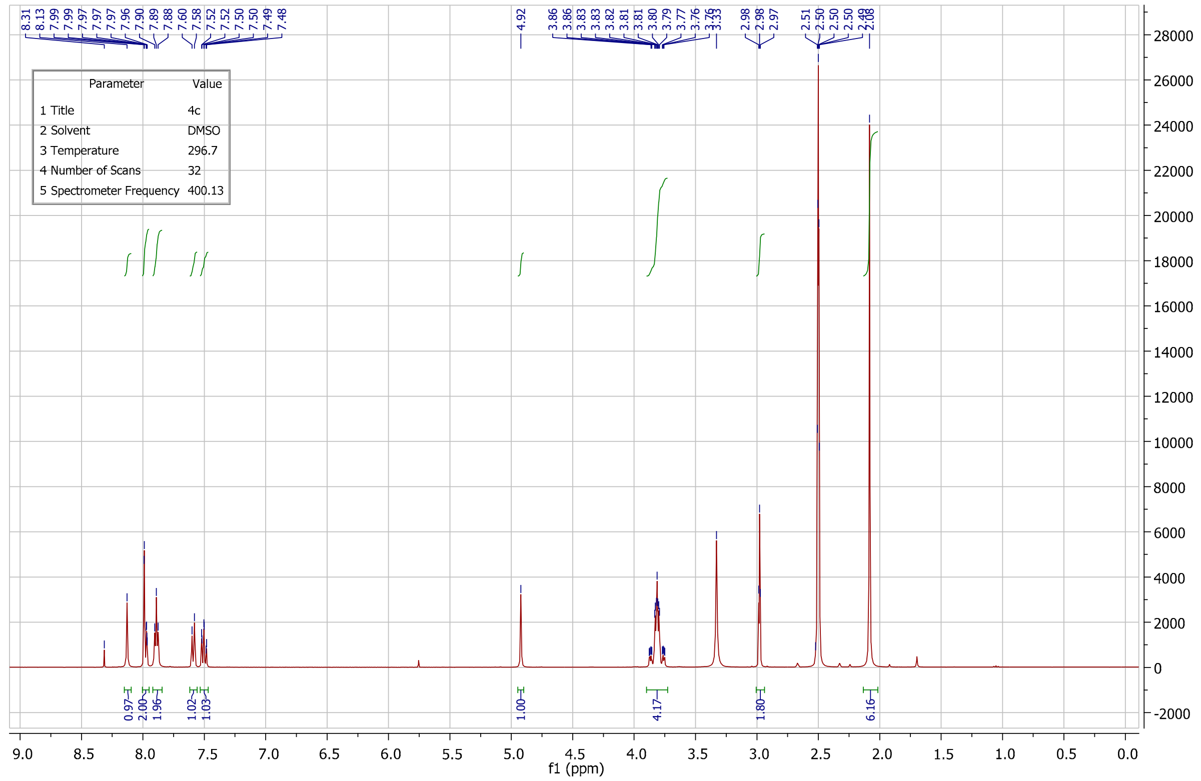

^{1}H NMR (400 MHz, DMSO) δ 8.13 (s, 1H), 8.01 – 7.93 (m, 2H), 7.89 (t, *J* = 5.6 Hz, 2H), 7.59 (d, *J* = 7.7 Hz, 1H), 7.50 (td, *J* = 7.7, 1.2 Hz, 1H), 4.92 (s, 1H), 3.89 – 3.74 (m, 4H), 2.98 (t, *J* = 2.4 Hz, 2H), 2.08 (s, 6H).

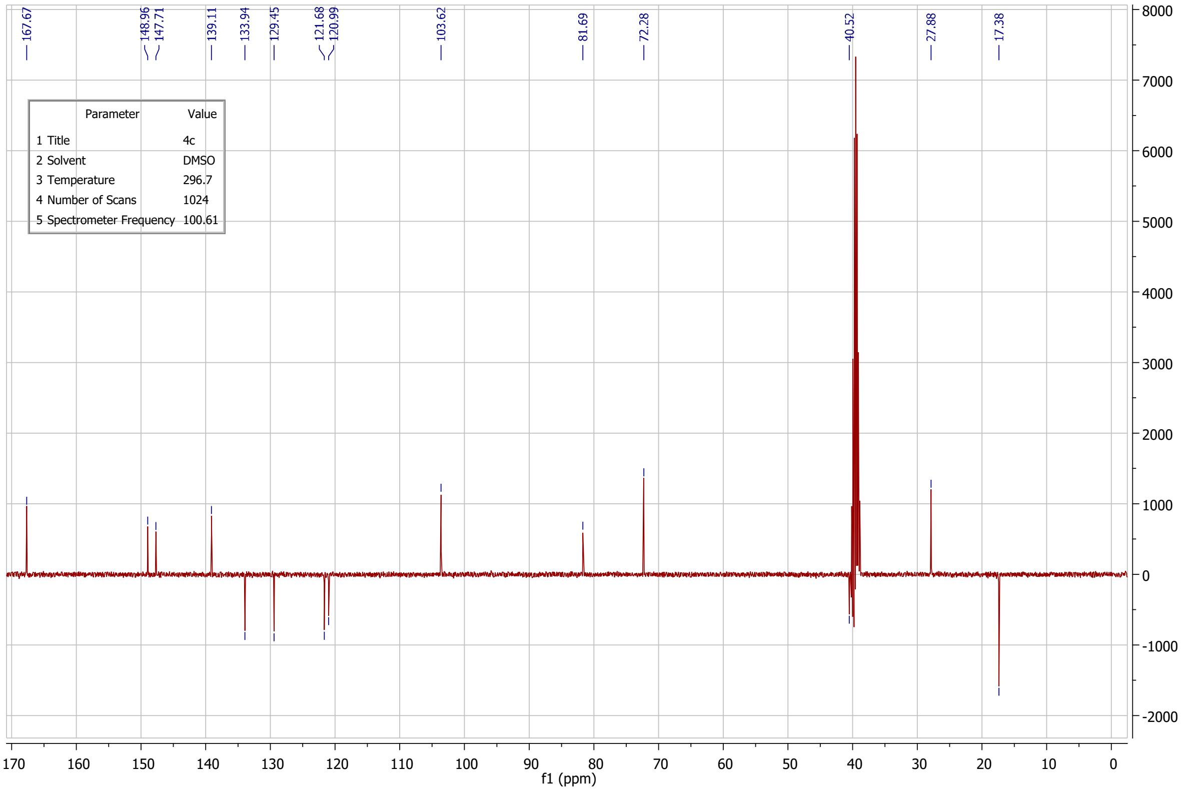

^{13}C NMR (101 MHz, DMSO) δ 167.67, 148.96, 147.71, 139.11, 133.94, 129.45, 121.68, 120.99, 103.62, 81.69, 72.28, 40.52, 27.88, 17.38.

Compound 4d:

4-(2,1,3-Benzoxadiazol-4-yl)-2,6-dimethyl-*N3*,*N5*-bis(prop-2-yn-1-yl)-1,4-dihydropyridine-3,5-dicarboxamide (4d).

Compound **4d** was prepared according to the general procedure from 2,1,3-benzoxadiazole-4-benzaldehyde (1 equiv., 0.72 mmol, 107 mg), 3-oxo-*N*-(prop-2-yn-1-yl)butanamide 2a (2 equiv., 1.47 mmol, 200 mg) and ammonium carbonate (1.2 equiv., 0.86 mmol, 83 mg) at 50 °C over 15 h. Then, the mixture was cooled to room temperature and precipitated 4d was filtered and resuspended in toluene. Solvent was removed under reduced pressure and the residue was washed with diethyl ether and filtered again to finally afford 141.5 mg (51%) of **4d** as dark yellowish powder.

^{1}H NMR (400 MHz, DMSO-d_6) δ 7 δ 8.15 (s, 1H), 7.87 – 7.73 (m, 3H), 7.46 (dd, *J* = 8.8, 6.8 Hz, 1H), 7.13 (d, *J* = 6.6 Hz, 1H), 5.23 (s, 1H), 3.90 – 3.67 (m, 4H), 2.96 (s, 2H), 2.03 (s, 6H).

^{13}C NMR (101 MHz, DMSO-d_6) δ 167.78, 149.51, 148.05, 138.95, 134.57, 132.92, 127.93, 113.41, 102.32, 81.64, 72.29, 27.88, 17.27.

Anal. Calcd. for $C_{21}H_{19}N_5O_5$: C, 64.77; H, 4.92; N, 17.98; found: C, 62.89; H, 4.88; N, 18.08.

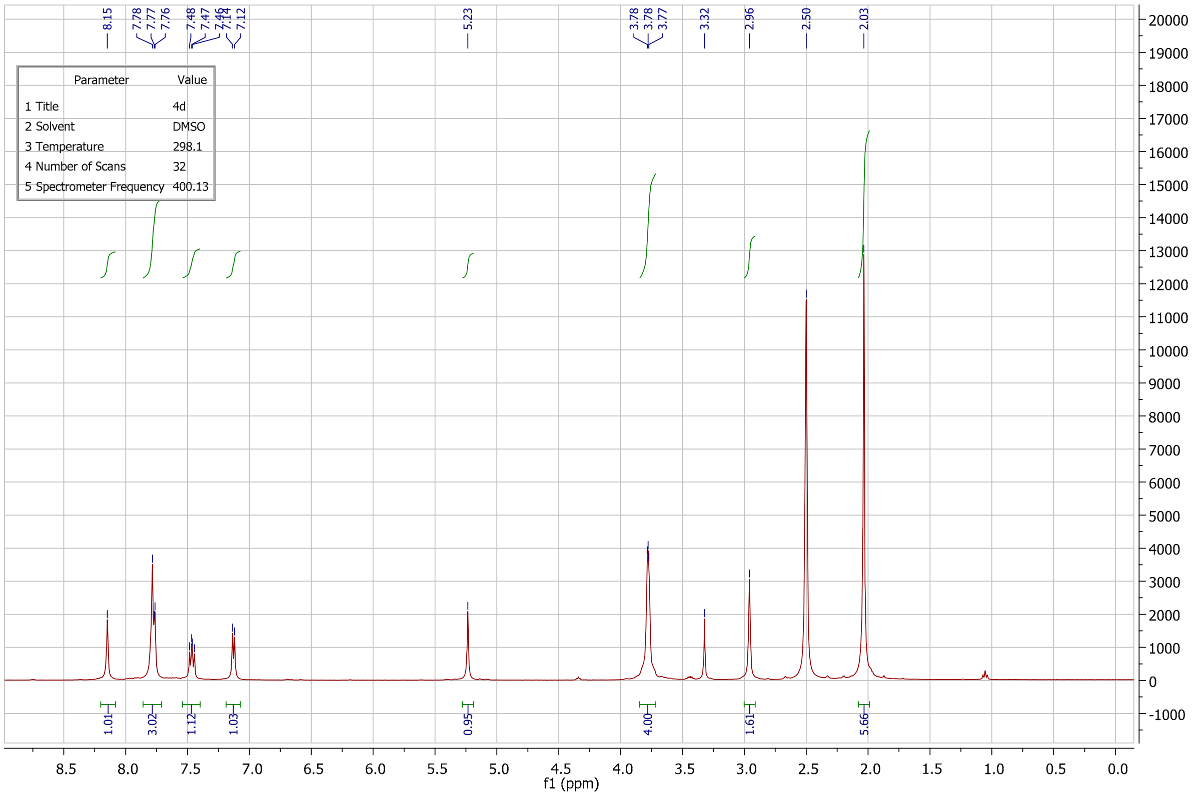

^{1}H NMR (400 MHz, DMSO) δ 8.15 (s, 1H), 7.87 – 7.73 (m, 3H), 7.46 (dd, J = 8.8, 6.8 Hz, 1H), 7.13 (d, J = 6.6 Hz, 1H), 5.23 (s, 1H), 3.90 – 3.67 (m, 4H), 2.96 (s, 2H), 2.03 (s, 6H).

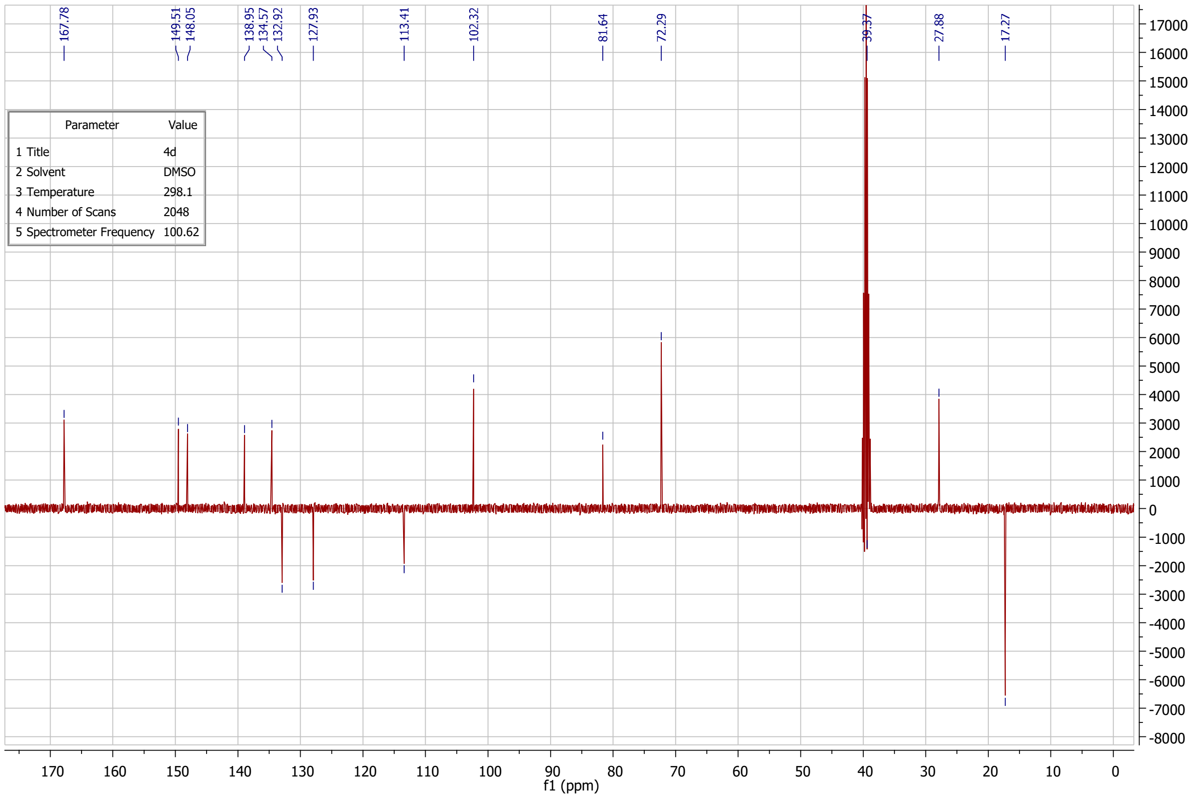

^{13}C NMR (101 MHz, DMSO) δ 167.78, 149.51, 148.05, 138.95, 134.57, 132.92, 127.93, 113.41, 102.32, 81.64, 72.29, 27.88, 17.27.

Compound 4e:

4-(2-Chlorophenyl)-2,6-dimethyl-*N3*,*N5*-bis(prop-2-yn-1-yl)-1,4-dihydropyridine-3,5-dicarboxamide.

This compound was prepared according to the general procedure starting from 2-chlorobenzaldehyde (1 equiv., 1.80 mmol, 0.20 mL), 3-oxo-*N*-(prop-2-yn-1-yl)butanamide 2a (2 equiv., 3.59 mmol, 500 mg) and ammonium carbonate (1.2 equiv., 2.16 mmol, 207 mg) at 45 °C over 15 h. Then, it was cooled to room temperature and extracted with CH_2Cl_2 three times. Organic layers were combined, dried over Na_2SO_4 and evaporated. The residue was washed with diethyl ether and filtered to afford 129 mg (56%) of 4e as white powder.

^{1}H NMR (400 MHz, DMSO-d_6) δ 7.96 (s, 1H), 7.72 (s, 2H), 7.28 (d, J = 7.3 Hz, 1H), 7.24 – 7.19 (m, 2H), 7.10 (t, J = 7.1 Hz, 1H), 5.10 (s, 1H), 3.82 (d, J = 2.7 Hz, 4H), 3.04 (s, 2H), 2.01 (s, 6H).

^{13}C NMR (101 MHz, DMSO-d_6) δ 167.98, 144.83, 137.89, 130.79, 130.16, 128.73, 127.61, 127.48, 104.47, 81.49, 72.69, 40.20, 27.89, 16.97.

Anal. Calcd. for $C_{21}H_{20}ClN_3O_2$: C, 66.05; H, 5.28; N, 11.00; found: C, 64.86; H, 5.31; N, 10.91.

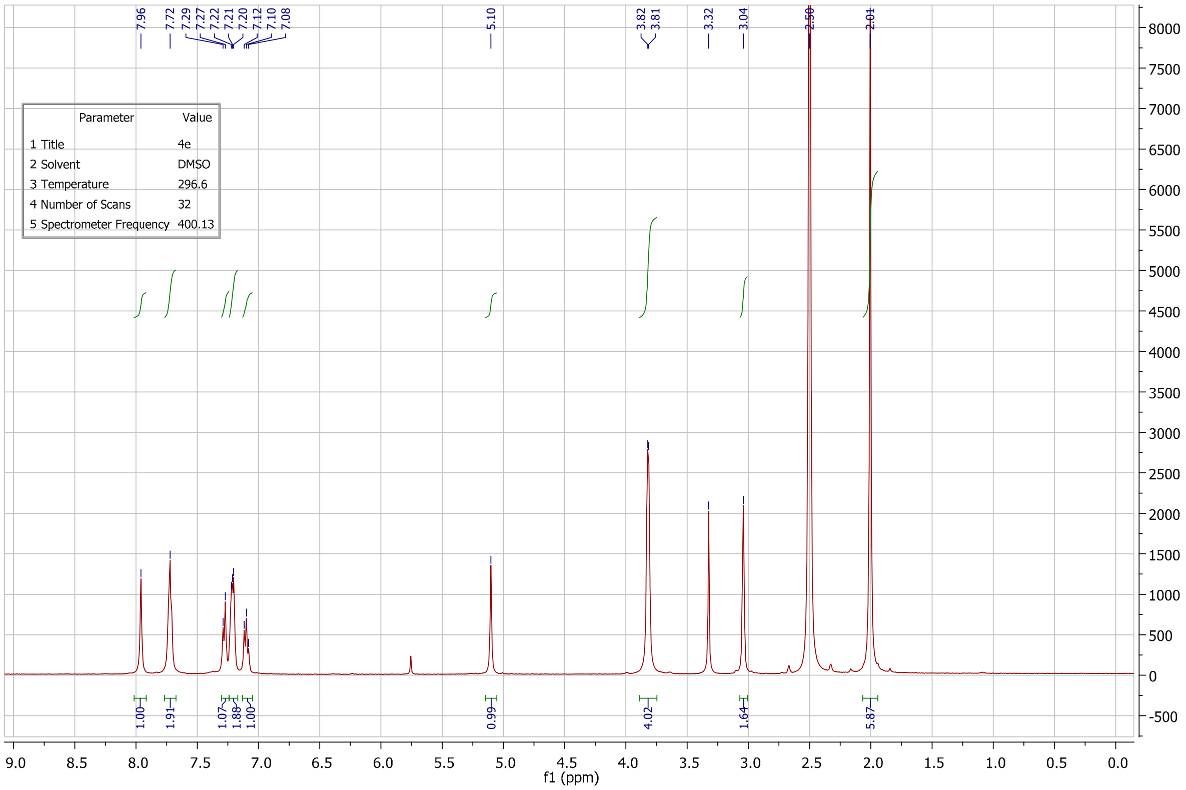

^{1}H NMR (400 MHz, DMSO) δ 7.96 (s, 1H), 7.72 (s, 2H), 7.28 (d, J = 7.3 Hz, 1H), 7.24 – 7.19 (m, 2H), 7.10 (t, J = 7.1 Hz, 1H), 5.10 (s, 1H), 3.82 (d, J = 2.7 Hz, 4H), 3.04 (s, 2H), 2.01 (s, 6H).

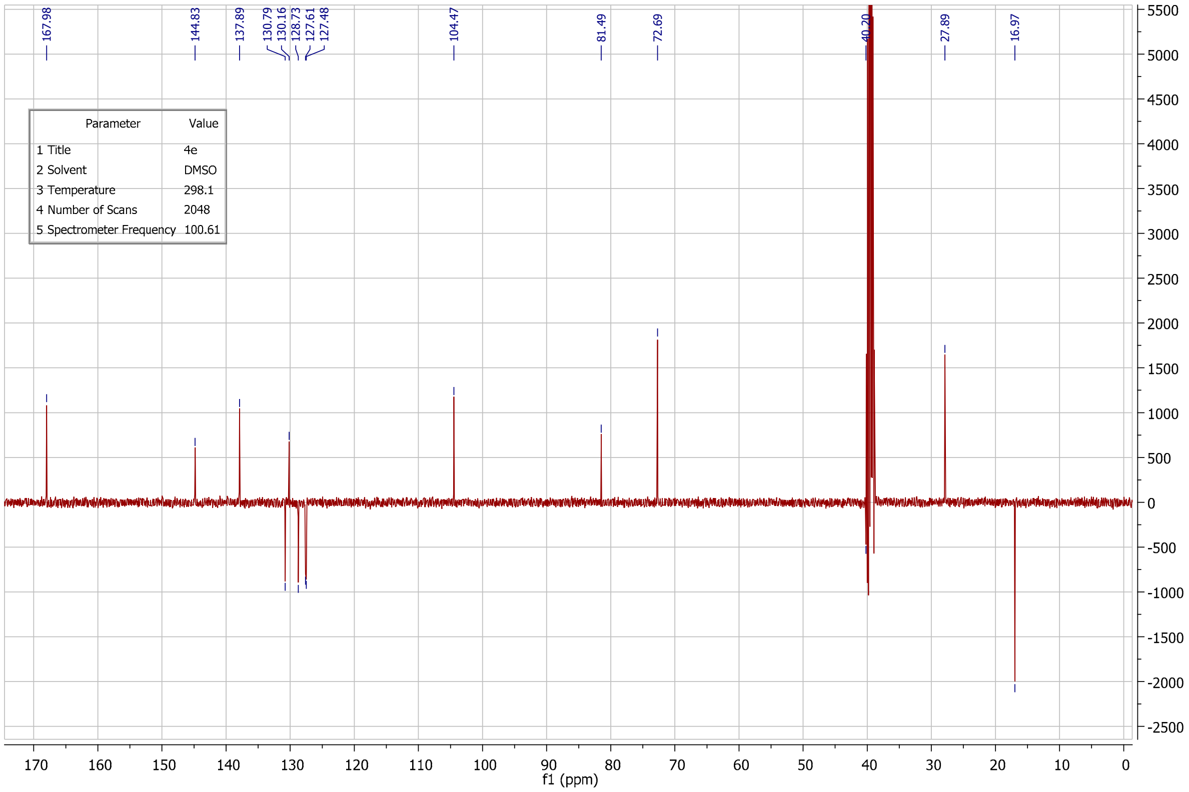

^{13}C NMR (101 MHz, DMSO) δ 167.98, 144.83, 137.89, 130.79, 130.16, 128.73, 127.61, 127.48, 104.47, 81.49, 72.69, 40.20, 27.89, 16.97.

Compound 4f:

4-(2-Methoxyphenyl)-2,6-dimethyl-*N3*,*N5*-bis(prop-2-yn-1-yl)-1,4-dihydropyridine-3,5-dicarboxamide.

Compound **4f** was prepared according to the general procedure starting from 2-methoxybenzaldehyde (1 equiv., 0.72 mmol, 0.087 mL), 3-oxo-N-(prop-2-yn-1-yl)butanamide **2a** (2 equiv., 1.44 mmol, 200 mg) and ammonium carbonate (1.7 equiv., 0.86 mmol, 119 mg) at 50 °C over 15 h. Then, it was cooled to room temperature and water was poured into the mixture. Precipitated **4f** was filtered and washed with diethyl ether to afford 78.7 mg (30%) as a light yellowish solid.

^{1}H NMR (400 MHz, DMSO-d_6) δ 8.05 (s, 1H), 7.42 (s, 2H), 7.20 – 7.05 (m, J = 16.1, 7.6 Hz, 3H), 6.86 (t, J = 8.2 Hz, 2H), 4.92 (s, 1H), 3.92 – 3.74 (m, 7H), 3.07 (s, 2H), 2.12 (s, 6H).

^{13}C NMR (101 MHz, DMSO-d_6) δ 167.47, 153.60, 139.92, 135.88, 129.43, 127.43, 121.29, 110.45, 104.13, 81.50, 72.80, 55.56, 32.69, 28.11, 17.55.

HRMS ESI-TOF [M]$^+$ m/z calcd. for $C_{22}H_{23}N_3O_3$: 377,1724, found: 377,1739.

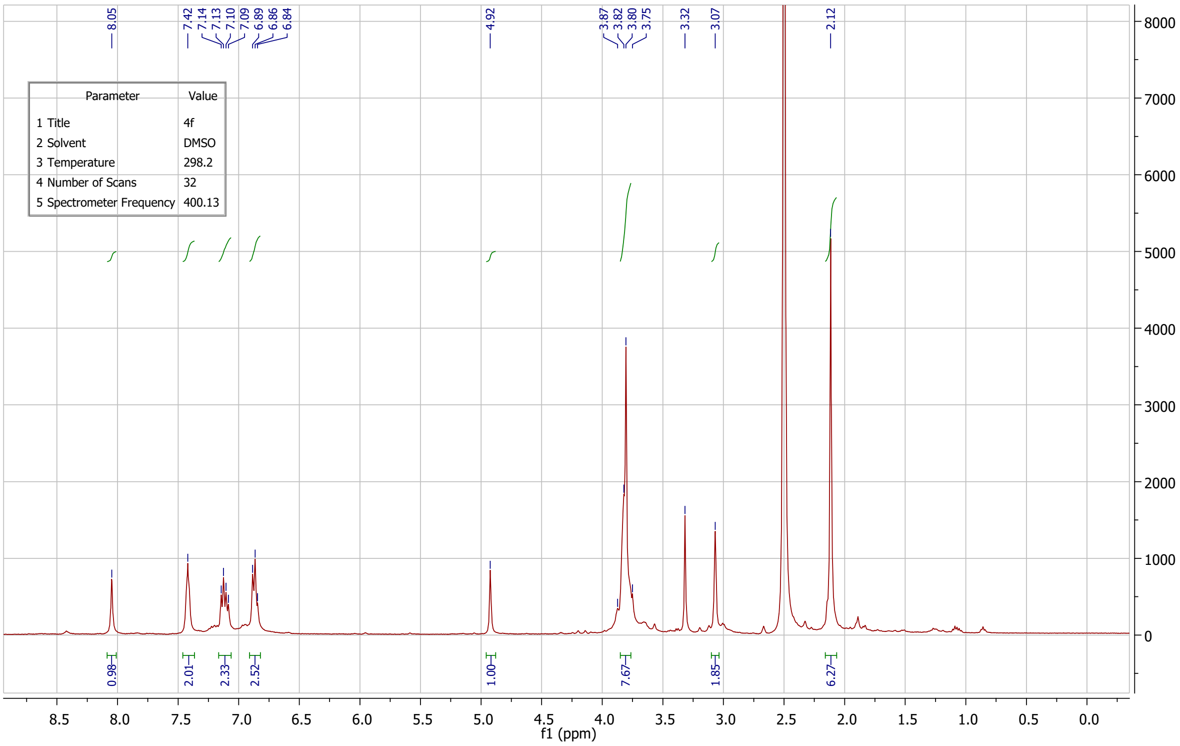

^{1}H NMR (400 MHz, DMSO) δ 8.05 (s, 1H), 7.42 (s, 2H), 7.20 – 7.05 (m, 3H), 6.86 (t, J = 8.2 Hz, 2H), 4.92 (s, 1H), 3.92 – 3.74 (m, 7H), 3.07 (s, 2H), 2.12 (s, 6H).

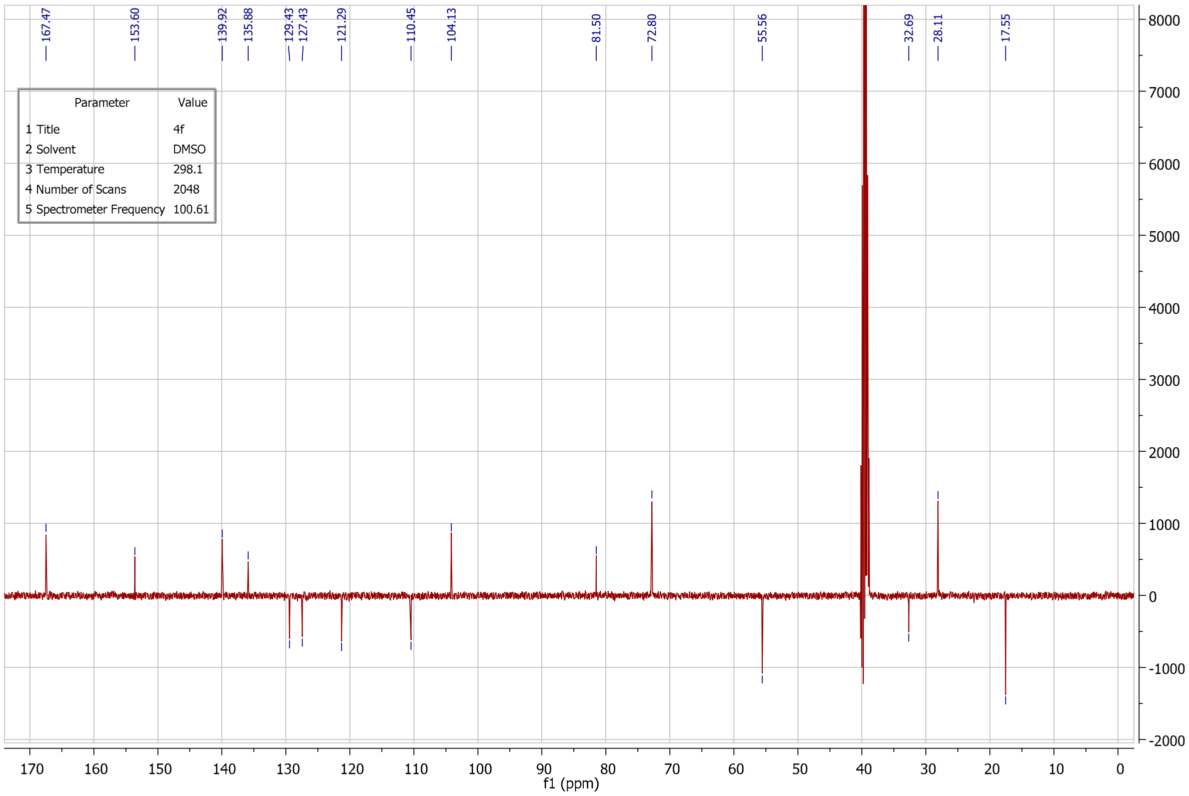

^{13}C NMR (101 MHz, DMSO) δ 167.47, 153.60, 139.92, 135.88, 129.43, 127.43, 121.29, 110.45, 104.13, 81.50, 72.80, 55.56, 32.69, 28.11, 17.55.

Compound 4g:

4-(2-Bromophenyl)-2,6-dimethyl-*N3*,*N5*-bis(prop-2-yn-1-yl)-1,4-dihydropyridine-3,5-dicarboxamide.

This compound was prepared according to the general procedure starting from 2-bromobenzaldehyde (1 equiv., 1.08 mmol, 0.126 mL), 3-oxo-*N*-(prop-2-yn-1-yl)butanamide **2a** (2 equiv., 2.16 mmol, 300 mg) and ammonium carbonate (1.2 equiv., 1.29 mmol, 124 mg) at 45 °C over 15 h. Precipitated 4g was filtered and washed with diethyl ether over 1 h to afford 154 mg (34%) as a white solid.

^{1}H NMR (400 MHz, DMSO-d_6) δ 7.96 (s, 1H), 7.71 (t, *J* = 5.1 Hz, 2H), 7.38 (d, *J* = 7.9 Hz, 1H), 7.32 – 7.19 (m, 2H), 7.09 – 6.96 (m, 1H), 5.07 (s, 1H), 3.95 – 3.75 (m, 4H), 3.05 (s, 2H), 2.00 (s, 6H).

^{13}C NMR (101 MHz, DMSO-d_6) δ 167.98, 146.90, 137.54, 132.00, 131.03, 128.18, 128.00, 120.49, 104.90, 81.47, 72.78, 41.59, 27.89, 16.91.

Anal. Calcd. for $C_{22}H_{23}BrN_3O_3$: C, 59.17; H, 4.73; N, 9.86; found: C, 58.79; H, 4.80; N, 9.88.

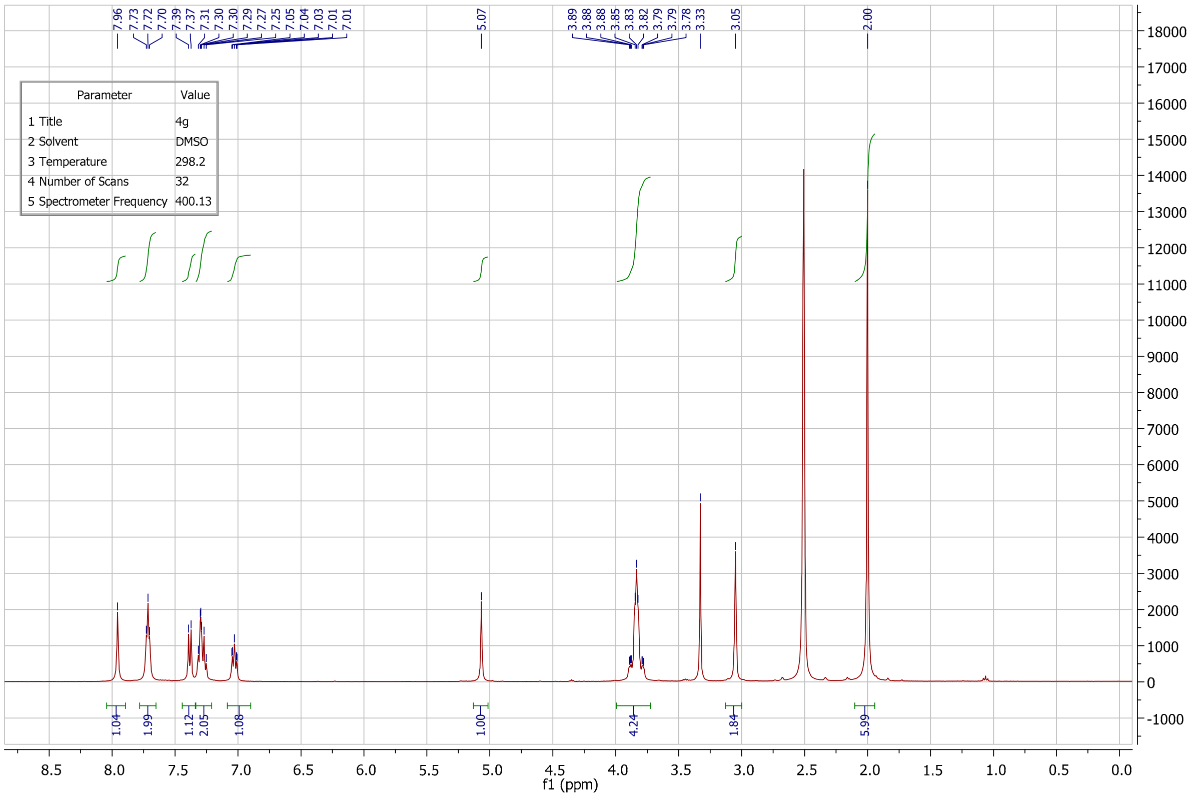

^{1}H NMR (400 MHz, DMSO δ 7.96 (s, 1H), 7.71 (t, *J* = 5.1 Hz, 2H), 7.38 (d, *J* = 7.9 Hz, 1H), 7.32 – 7.19 (m, 2H), 7.09 – 6.96 (m, 1H), 5.07 (s, 1H), 3.95 – 3.75 (m, 4H), 3.05 (s, 2H), 2.00 (s, 6H).

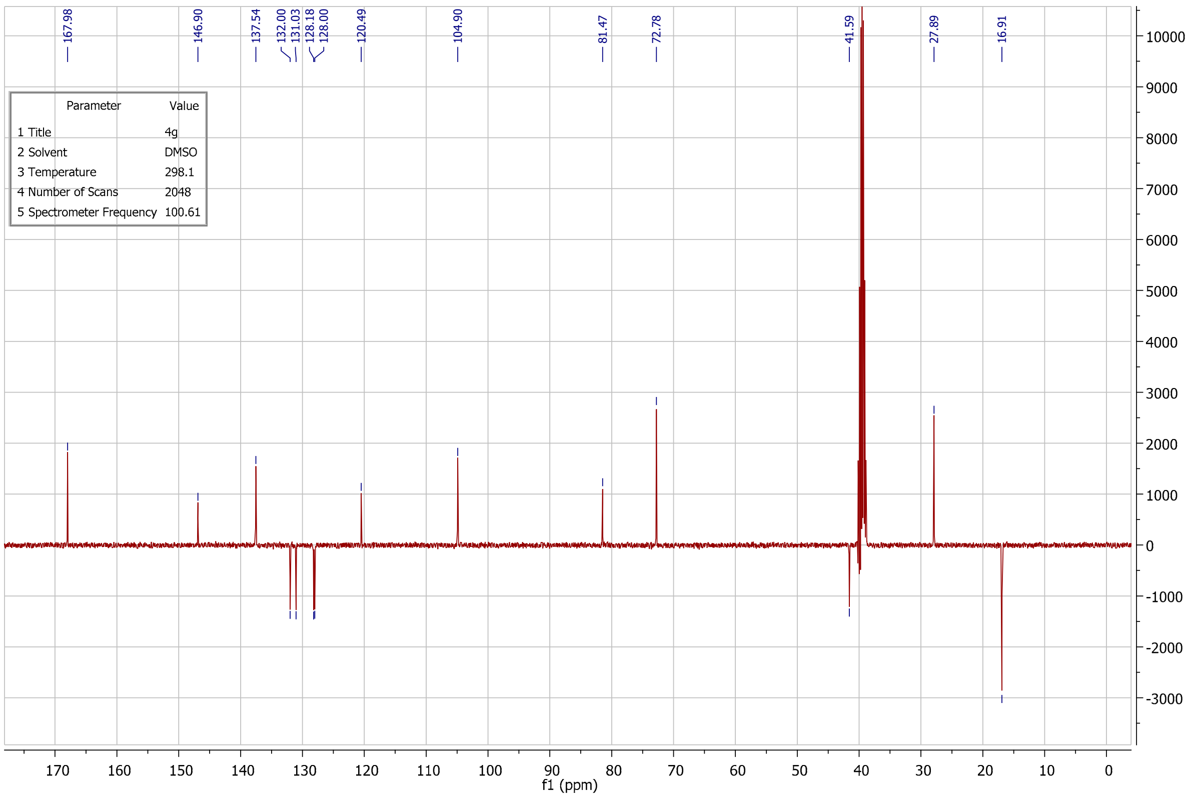

^{13}C NMR (101 MHz, DMSO) δ 167.98, 146.90, 137.54, 132.00, 131.03, 128.18, 128.00, 120.49, 104.90, 81.47, 72.78, 41.59, 27.89, 16.91.

Compound 4h:

4-(3-Bromophenyl)-2,6-dimethyl-*N3,N5*-bis(prop-2-yn-1-yl)-1,4-dihydropyridine-3,5-dicarboxamide.

Compound **4h** was prepared according to the general procedure starting from 2-bromobenzaldehyde (1 equiv., 1.08 mmol, 0.126 mL), 3-oxo-N-(prop-2-yn-1-yl)butanamide **2a** (2 equiv., 2.157 mmol, 300 mg) and ammonium carbonate (1.2 equiv., 1.29 mmol, 124 mg) at 45 °C over 15 h. Precipitated **4h** was filtered and washed with diethyl ether over 1h to afford 56.8 mg (12%) as a white solid.

^{1}H NMR (400 MHz, DMSO-d_6) δ 8.03 (s, 1H), 7.80 (t, *J* = 5.1 Hz, 2H), 7.33 – 7.25 (m, 2H), 7.20 – 7.07 (m, 2H), 4.77 (s, 1H), 3.84 (qd, *J* = 17.3, 3.1 Hz, 4H), 3.02 (s, 2H), 2.07 (s, 6H).

^{13}C NMR (101 MHz, DMSO-d_6) δ 167.82, 149.33, 139.00, 130.21, 129.64, 128.76, 126.03, 121.56, 103.68, 81.74, 72.42, 40.34, 27.87, 17.36.

Anal. Calcd. for $C_{22}H_{23}BrN_3O_3$: C, 59.17; H, 4.73; N, 9.86; found: C, 57.88; H, 4.69; N, 9.78.

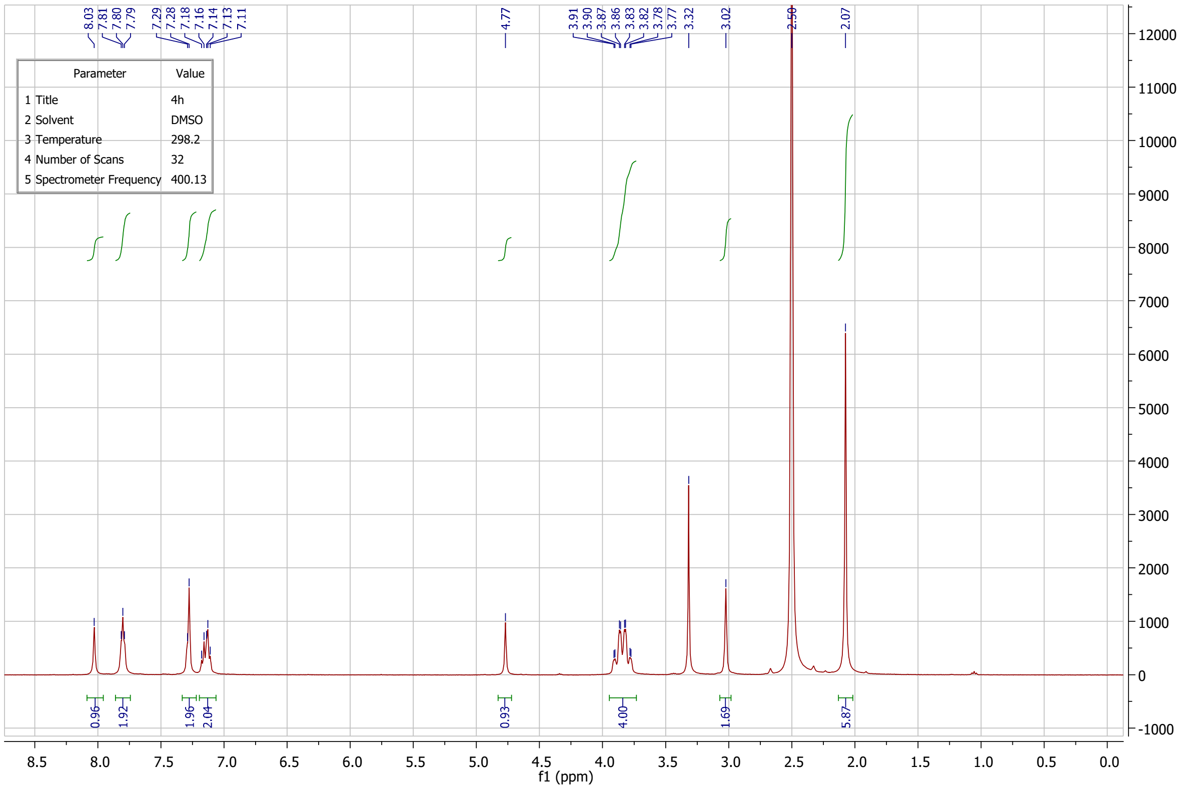

^{1}H NMR (400 MHz, DMSO) δ 8.03 (s, 1H), 7.80 (t, J = 5.1 Hz, 2H), 7.33 – 7.25 (m, 2H), 7.20 – 7.07 (m, 2H), 4.77 (s, 1H), 3.84 (qd, J = 17.3, 3.1 Hz, 4H), 3.02 (s, 2H), 2.07 (s, 6H).

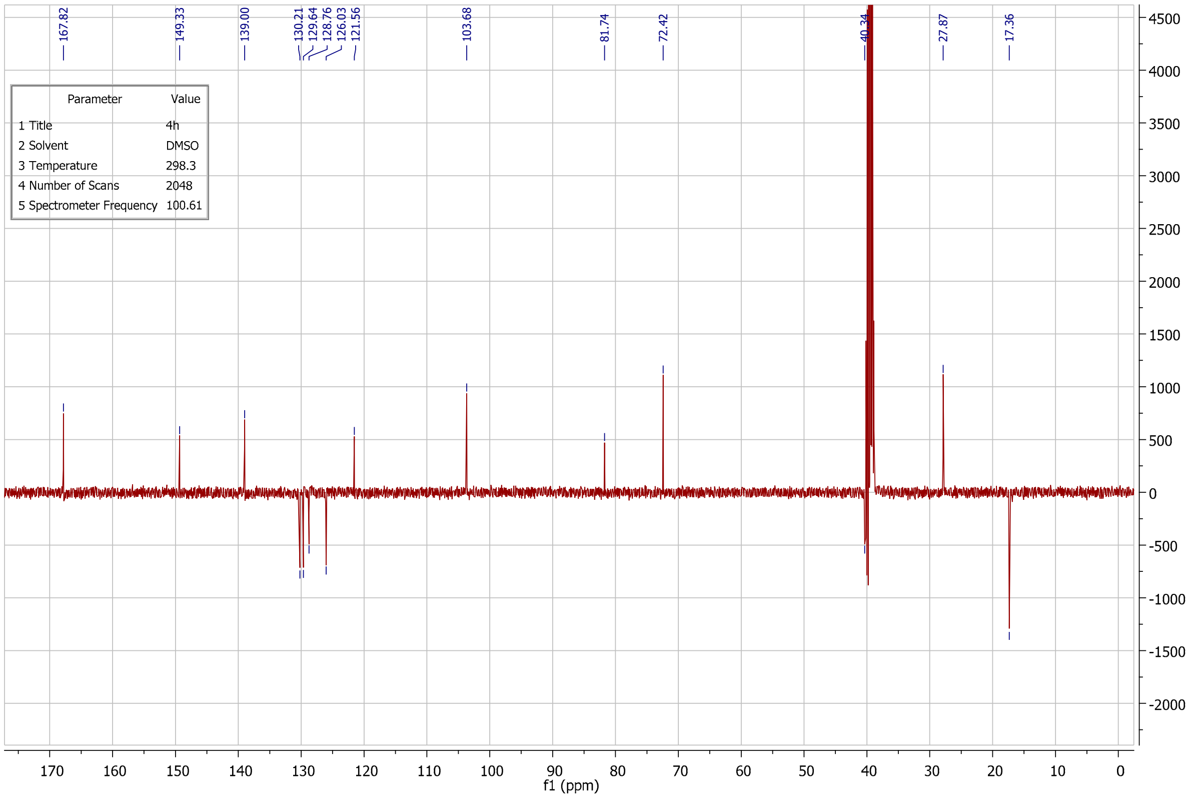

^{13}C NMR (101 MHz, DMSO) δ 167.82, 149.33, 139.00, 130.21, 129.64, 128.76, 126.03, 121.56, 103.68, 81.74, 72.42, 40.34, 27.87, 17.36.

Compound 4i:

4-(2-Chlorophenyl)-*N*2,*N*3,5,6-tetramethyl-*N*3,*N*5-bis(prop-2-yn-1-yl)-1,4-dihydropyridine-3,5-dicarboxamide.

This compound was prepared according to the general procedure from 2-chlorobenzaldehyde (1 equiv., 1.09 mmol, 0.126 mL), *N*-methyl-3-oxo-*N*-(prop-2-yn-1-yl)butanamide **2b** (3 equiv., 3.27 mmol, 500 mg) and ammonium carbonate (1.2 equiv., 1.96 mmol, 188 mg) at 55 °C over 19 h. CH_2Cl_2 was added to the mixture. The organic layer was washed with water, brine, then dried over Na_2SO_4 and evaporated to finally be purified by flash column chromatography using 25/75 hexane/EtOAc + 1% Et_3N as eluent. 4i was washed with pentane:diethyl ether 1:1 v/v and filtered to finally afford 95 mg (12%) as a light beige solid.

^{1}H NMR (400 MHz, DMSO-*d*$_6$) δ 7.78 (s, 1H), 7.38 (bs, 1H), 7.29 – 7.19 (m, 2H), 7.16 – 7.07 (m, 1H), 4.93 (s, 1H), 4.00 (t, *J* = 19.3 Hz, 4H), 3.13 (s, 2H), 2.78 (s, 6H), 1.74 (s, 6H).

^{13}C NMR (101 MHz, DMSO-*d*$_6$) δ 169.92, 133.23, 131.20, 127.80, 127.32, 79.15, 74.33, 15.94.

Anal. Calcd. for $C_{23}H_{24}ClN_3O_2$: C, 67.39; H, 5.90; N, 10.25; found: C, 66.12; H, 5.98; N, 10.30.

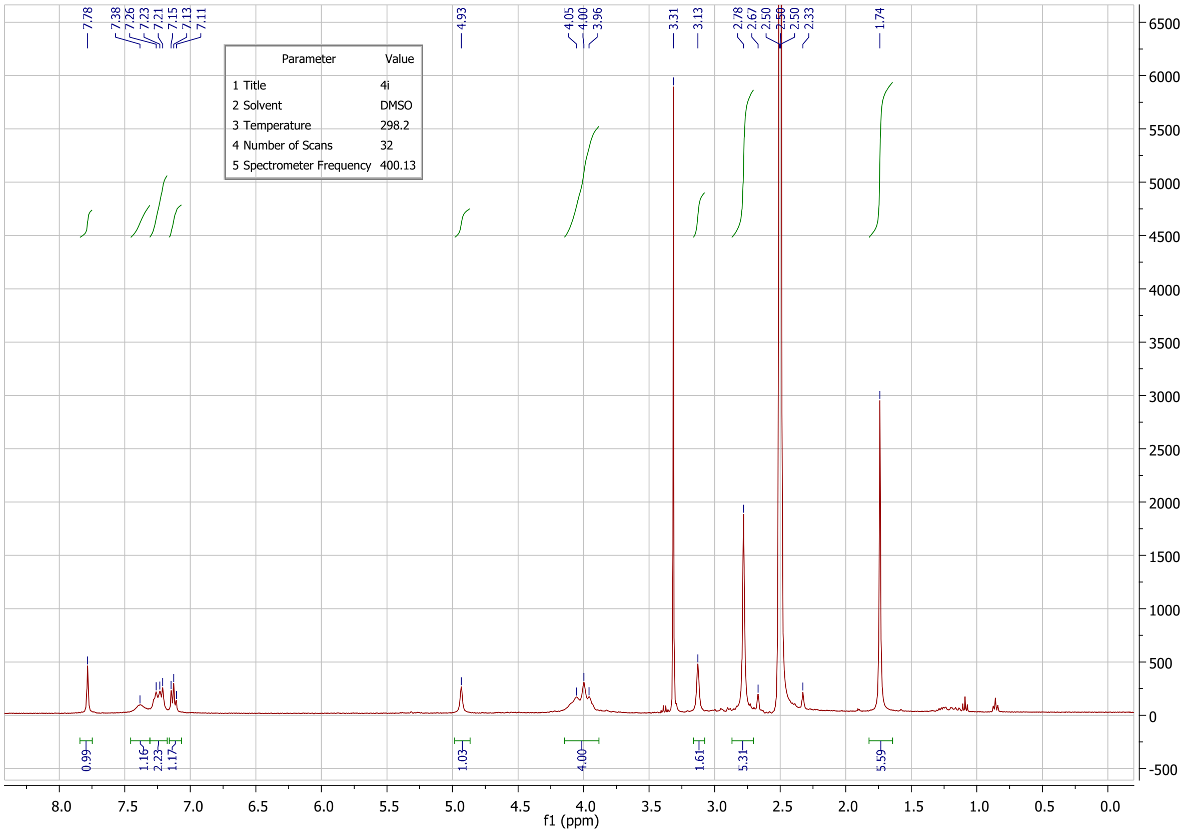

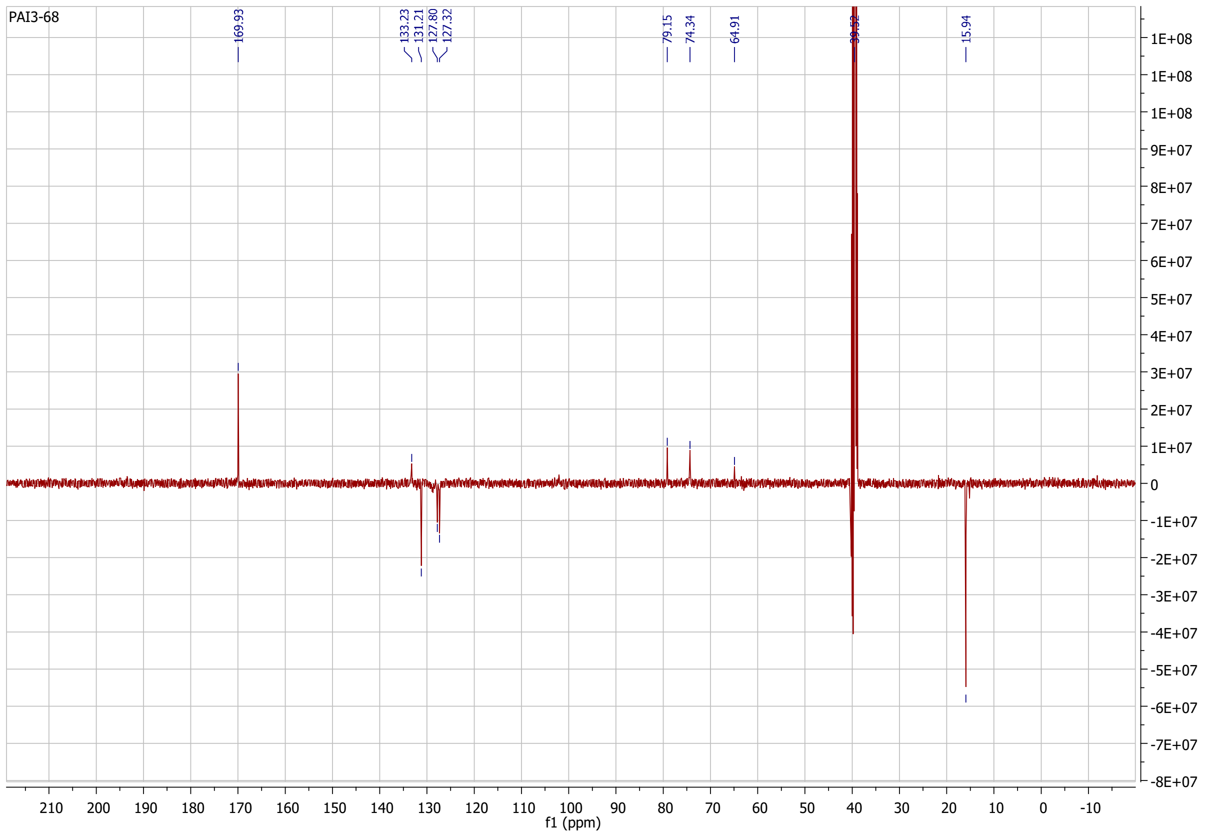

HPLC analysis of compound 4i was performed using a Avantor Chromaster instrument equipped with a L2400 Lachrom Elite DAD detector and a BDS HYPERSIL C18 column (5 µm – 250*4.6 mm). Peaks were detected at 205 nm and the system was operated at 40°C with a flow rate of 1.5 mL/min. The mobile phase was an isocratic mixture of methanol and water (4:6, v/v) containing 0.1% (w/v) of monopotassium phosphate and 0.05% (w/v) of phosphoric acid.

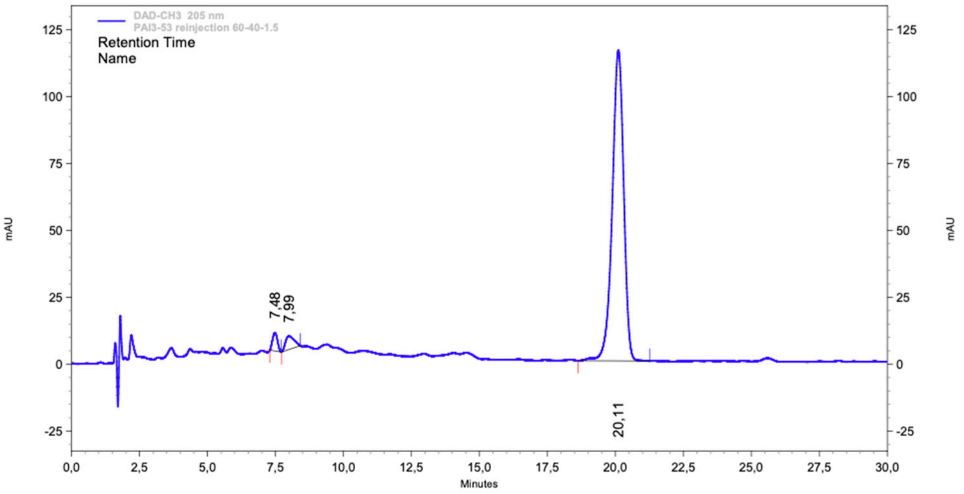

DAD-CH3 205 nm
Results

N°	Temps (min)	Surface	Int. code	Area Percent
1	7,48	346113	vv	2,41
2	7,99	444900	vv	3,09
3	20,11	13594259	vv	94,50

Compound 4j:

4-(2,3-Dichlorophenyl)-*N*2,*N*3,5,6-tetramethyl-*N*3,*N*5-bis(prop-2-yn-1-yl)-1,4-dihydropyridine-3,5-dicarboxamide.

Compound **4j** was prepared according to the general procedure starting from 2,3-dichlorobenzaldehyde (1 equiv., 1.14 mmol, 200 mg), *N*-methyl-3-oxo-*N*-(prop-2-yn-1-yl)butanamide 2b (2 equiv., 2.29 mmol, 350 mg) and ammonium carbonate (1.2 equiv., 1.37 mmol, 140 mg) at 45 °C over 19 h. Diethyl ether was added to the reaction crude. The organic layer was washed with water, brine, then dried over Na_2SO_4, and evaporated under reduced pressure to finally be purified by flash column chromatography using 94/6 CH_2Cl_2/MeOH + 1% NH_3 as eluent. 4j was washed with pentane:diethyl ether 1:1 v/v and filtered to finally afford 21.3 mg (7%) as light yellow powder.

^{1}H NMR (400 MHz, DMSO-d_6) δ 7.88 (s, 1H), 7.44 – 7.34 (m, 2H), 7.30 (d, *J* = 6.7 Hz, 1H), 4.99 (s, 1H), 4.05 (dd, *J* = 30.9, 17.2 Hz, 4H), 3.13 (s, 2H), 2.81 (s, 6H), 1.74 (s, 6H).

^{13}C NMR (101 MHz, DMSO-d_6) δ 169.72, 133.66, 129.72, 128.33, 128.11, 101.86, 74.33, 15.95, 13.90.

HRMS ESI-TOF [M]$^+$ m/z calcd. for $C_{23}H_{23}Cl_2N_3O_2$: 443,1149, found: 443,1167.

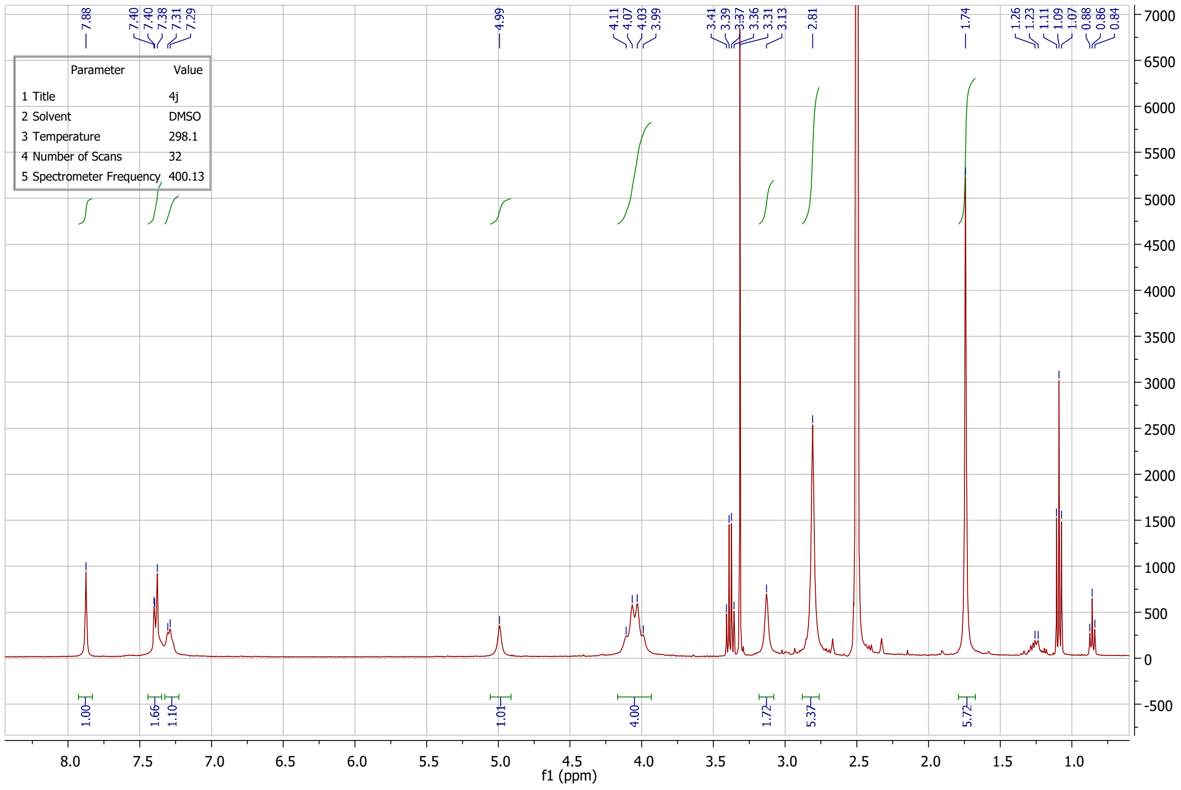

^{1}H NMR (400 MHz, DMSO δ 7.88 (s, 1H), 7.44 – 7.34 (m, 2H), 7.30 (d, 6.7 Hz, 1H), 4.99 (s, 1H), 4.05 (dd, = 30.9, 17.2 Hz, 4H), 3.13 (s, 2H), 2.81 (s, 6H), 1.74 (s, 6H).

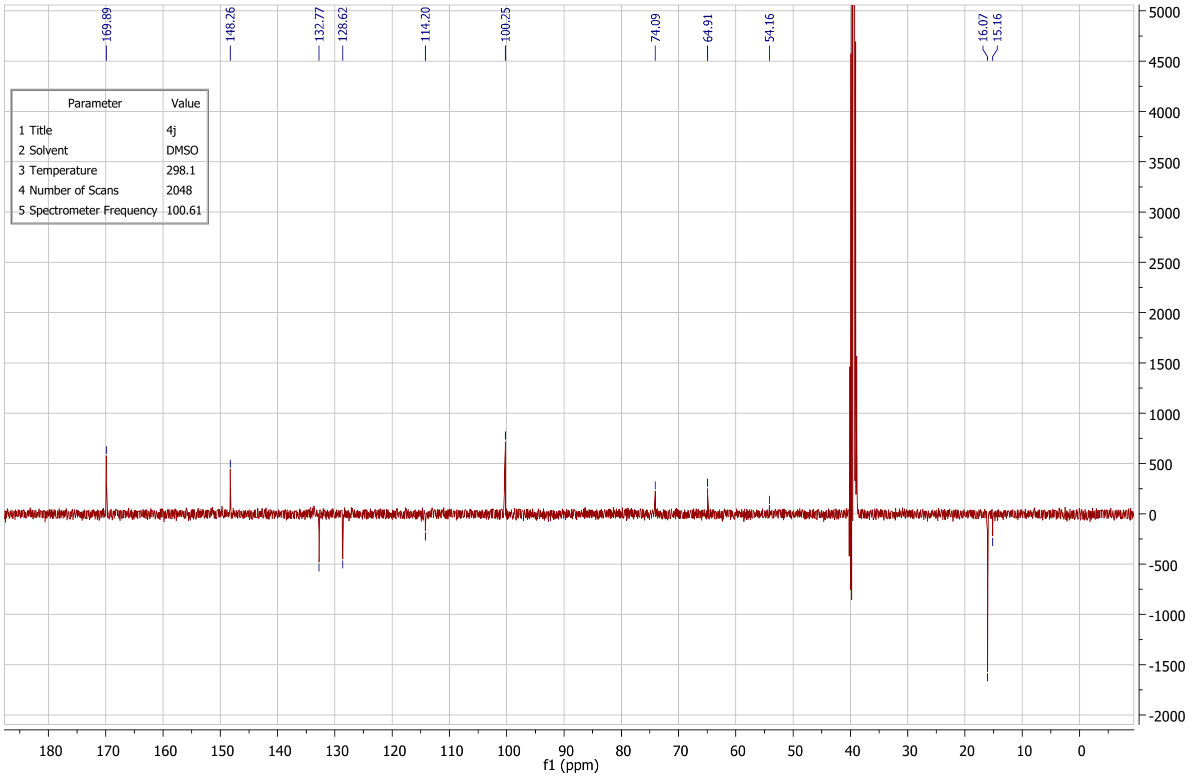
169.89
148.26
132.77
128.62
114.20
100.25
74.09
64.91
54.16
16.07
15.16
Parameter | Value
1 Title | 4j
2 Solvent | DMSO
3 Temperature | 298.1
4 Number of Scans | 2048
5 Spectrometer Frequency | 100.61
f1 (ppm)

Compound 4k:

4-(2-Methoxyphenyl)-*N*2,*N*3,5,6-tetramethyl-*N*3,*N*5-bis(prop-2-yn-1-yl)-1,4-dihydropyridine-3,5-dicarboxamide.

This compound was prepared according to the general procedure starting from 2-methoxybenzaldehyde (1 equiv., 1.63 mmol, 0.197 mL), *N*-methyl-3-oxo-*N*-(prop-2-yn-1-yl)butanamide **2b** (2 equiv., 3.27 mmol, 500 mg) and ammonium carbonate (1.2 equiv., 1.96 mmol, 188 mg) at 40 °C over 15 h. Diethyl ether was added to the reaction crude. The organic layer was washed with water, brine, then dried over Na_2SO_4, and reduced under pressure conditions to finally be purified by flash column chromatography using 96/4 CH_2Cl_2/MeOH + 1% NH_3 as eluent. 4k was washed with pentane:diethyl ether 1:1 v/v over 1 h and filtered to finally afford 45.6 mg (7%) as light beige powder.

^{1}H NMR (400 MHz, DMSO-d_6) δ 7.60 (s, 1H), 7.18 – 7.04 (m, 2H), 6.83 (dd, *J* = 16.3, 7.9 Hz, 2H), 4.78 (s, 1H), 4.01 (t, *J* = 20.9 Hz, 4H), 3.62 (s, 3H), 3.15 (s, 2H), 2.76 (s, 6H), 1.73 (s, 6H).

^{13}C NMR (101 MHz, DMSO-d_6) δ 170.58, 129.27, 127.22, 120.25, 110.47, 102.15, 94.29, 79.49, 74.06, 64.90, 55.20, 46.98, 34.46, 15.93.

HRMS ESI-TOF [M]$^+$ m/z calcd. for $C_{24}H_{27}N_3O_3$: 405,2042, found: 405,2052.

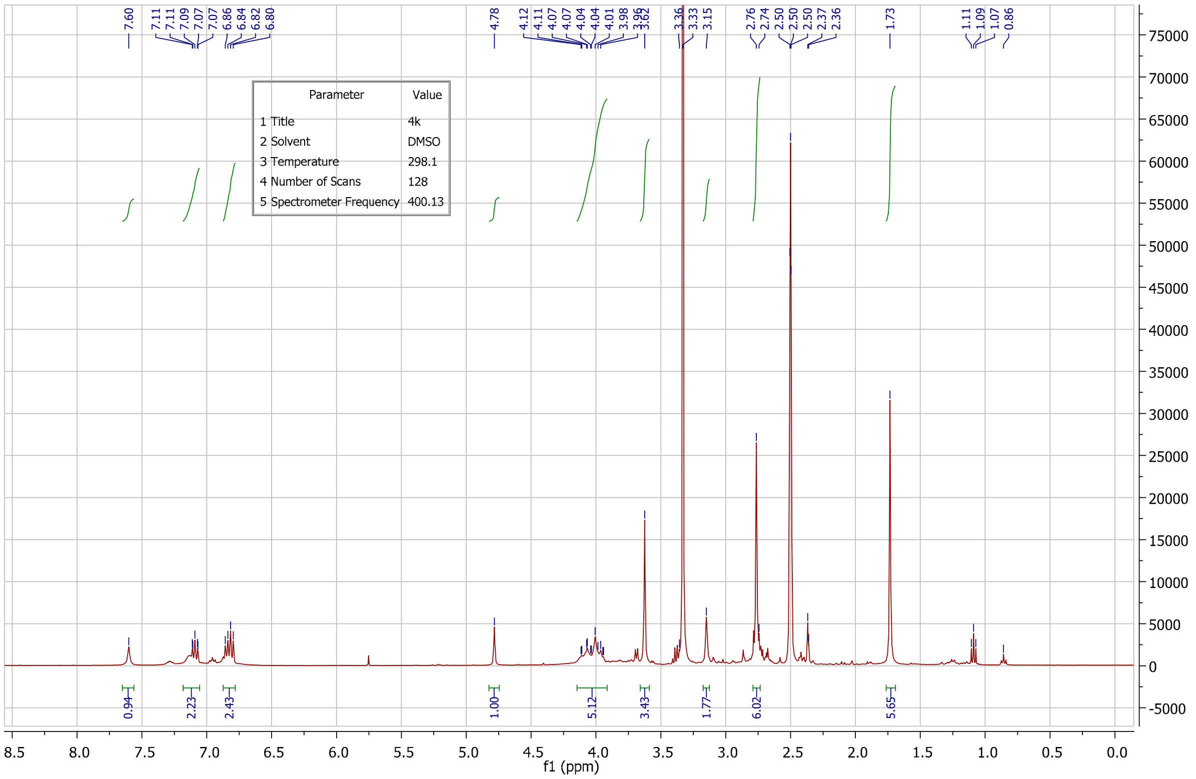

^{1}H NMR (400 MHz, DMSO) δ 7.60 (s, 1H), 7.13 – 7.06 (m, 2H), 6.83 (dd, *J* = 16.5, 8.0 Hz, 3H), 4.78 (s, 1H), 4.15 – 3.92 (m, 5H), 3.62 (s, 3H), 3.15 (s, 2H), 2.76 (s, 6H).

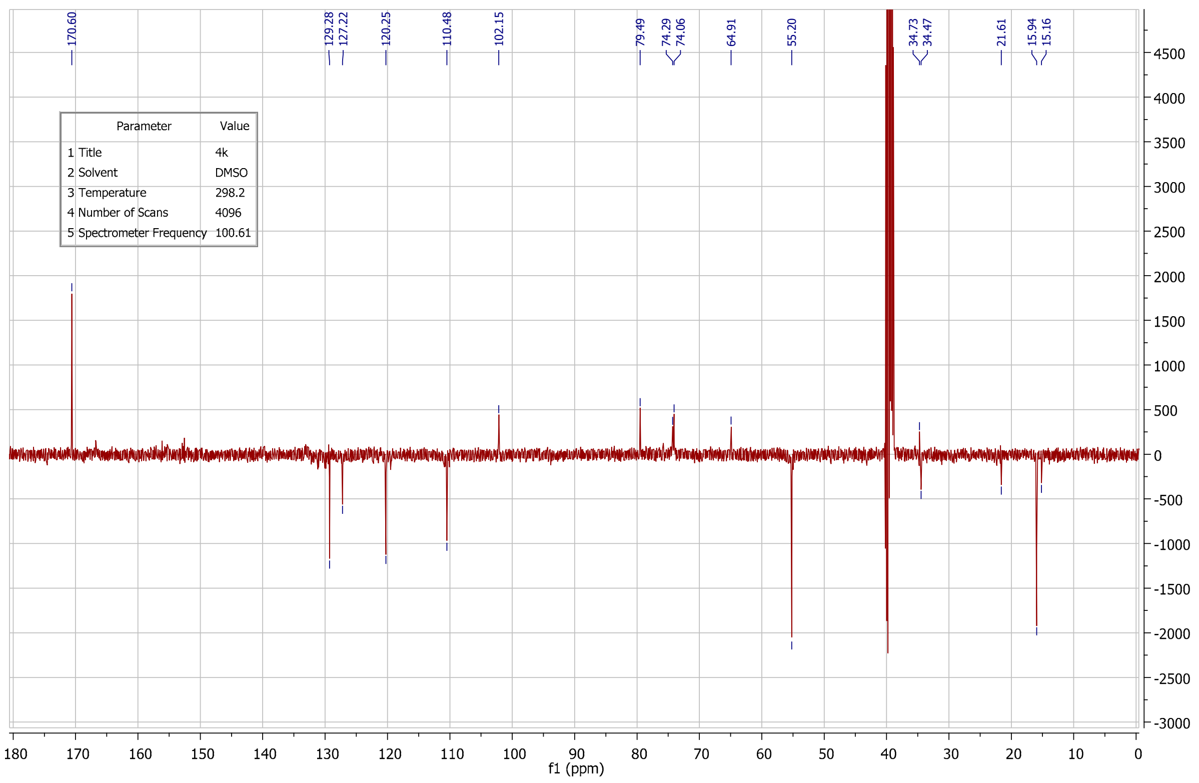

^{13}C NMR (101 MHz, DMSO) δ 170.60, 129.28, 127.22, 120.25, 110.48, 102.15, 79.49, 74.29, 74.06, 64.91, 55.20, 34.73, 34.47, 21.61, 15.94, 15.16.

Compound 4l:

4-(3-Methoxyphenyl)-*N2*,*N3*,5,6-tetramethyl-*N3*,*N5*-bis(prop-2-yn-1-yl)-1,4-dihydropyridine-3,5-dicarboxamide.

Compound **4l** was prepared according to the general procedure starting from 3-methoxybenzaldehyde (1 equiv., 1.63 mmol, 0.197 mL), *N*-methyl-3-oxo-*N*-(prop-2-yn-1-yl)butanamide 2b (2 equiv., 3.27 mmol, 500 mg) and ammonium carbonate (1.2 equiv., 1.96 mmol, 188 mg) at 45 °C over 15 h. CH_2Cl_2 was added to the reaction mixture. The organic layer was washed with water, brine, then dried over Na_2SO_4 and evaporated to finally be purified by flash column chromatography using 96/4 CH_2Cl_2/MeOH + 1% NH_3 as eluent. **4l** was washed with pentane:diethyl ether 1:1 v/v over 1 h and filtered to finally afford 56.5 mg (5%) as light beige powder.

^{1}H NMR (400 MHz, DMSO-d_6) δ δ 7.66 (s, 1H), 7.12 (t, *J* = 7.6 Hz, 1H), 6.67 (dd, *J* = 24.2, 7.4 Hz, 2H), 6.55 (s, 1H), 4.44 (s, 1H), 4.00 (s, 4H), 3.67 (s, 3H), 3.14 (s, 2H), 2.72 (s, 6H), 1.73 (s, 6H).

^{13}C NMR (101 MHz, DMSO-d_6) δ 170.48, 159.14, 146.53, 129.15, 119.76, 113.00, 111.48, 102.62, 79.26, 54.84, 44.85, 16.01.

HRMS ESI-TOF [M]$^+$ m/z calcd. for $C_{24}H_{27}N_3O_3$: 405.2040, found: 405.2052.

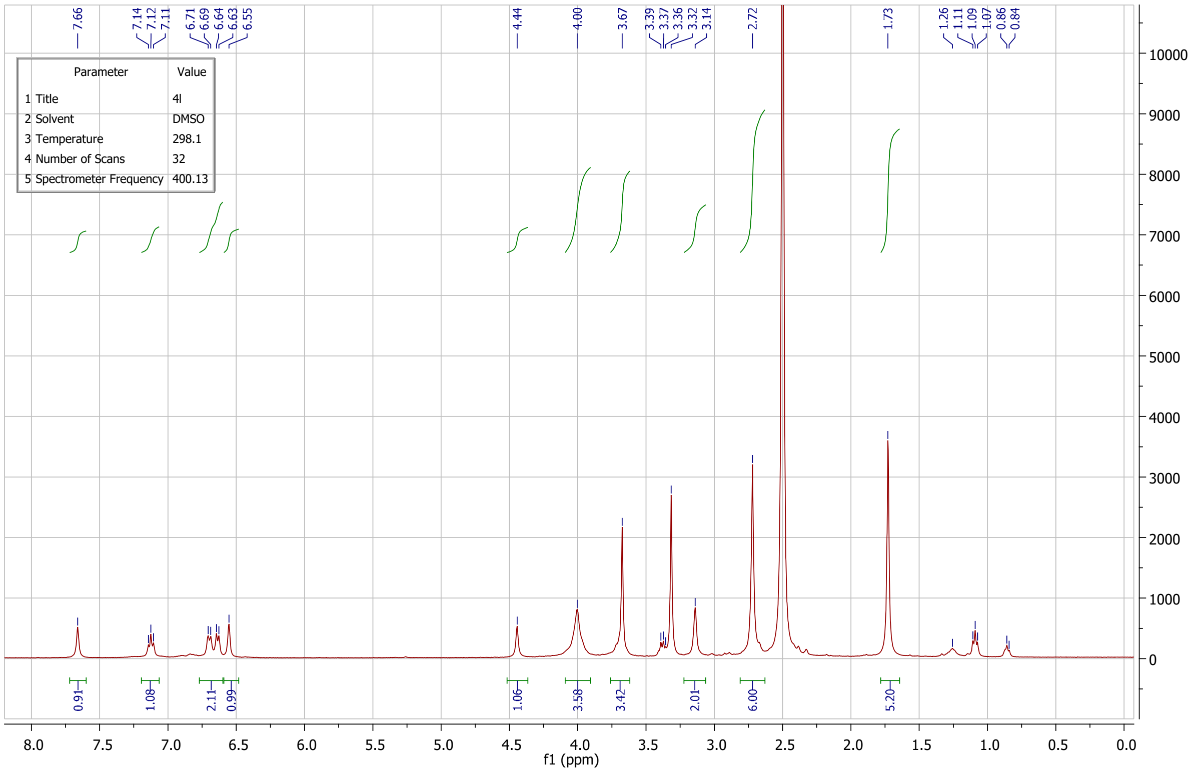

^{1}H NMR (400 MHz, DMSO) δ 7.66 (s, 1H), 7.12 (t, *J* = 7.6 Hz, 1H), 6.67 (dd, *J* = 24.2, 7.4 Hz, 2H), 6.55 (s, 1H), 4.44 (s, 1H), 4.00 (s, 4H), 3.67 (s, 3H), 3.14 (s, 2H), 2.72 (s, 6H), 1.73 (s, 6H).

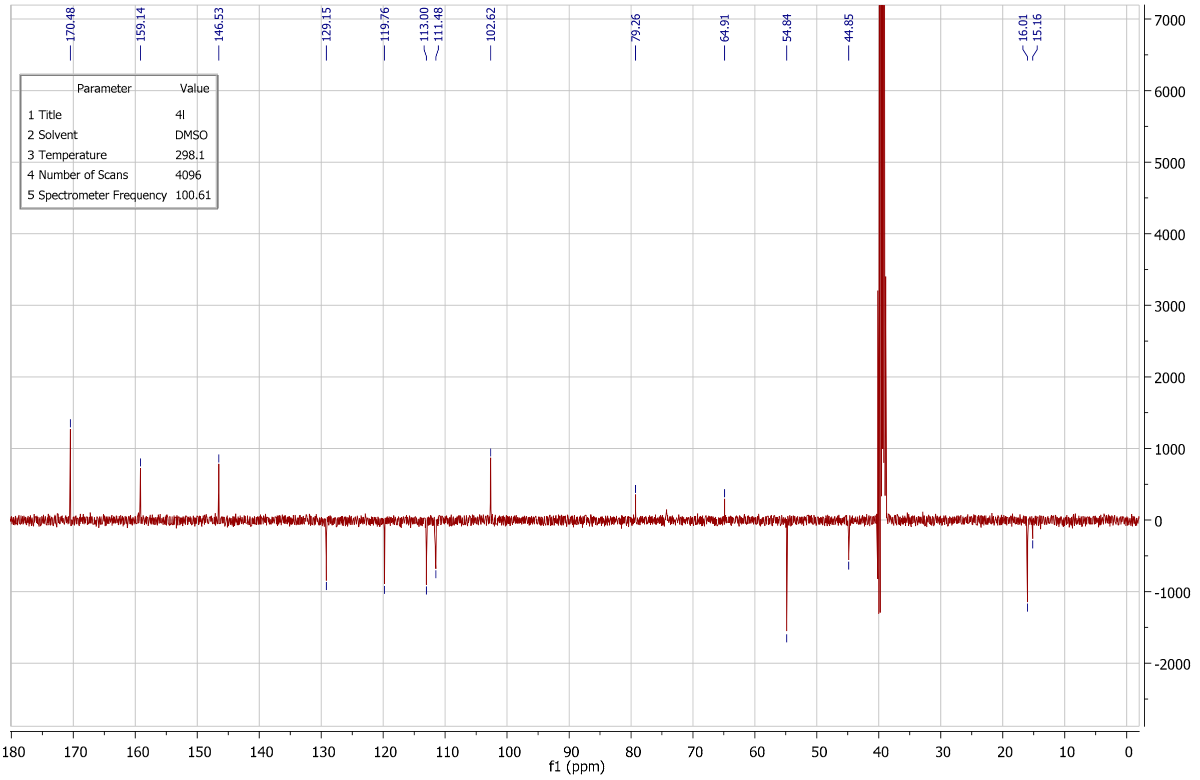

^{13}C NMR (101 MHz, DMSO) δ 170.48, 159.14, 146.53, 129.15, 119.76, 113.00, 111.48, 102.62, 79.26, 54.84, 44.85, 16.01.
